# The p.P888L SAP97 polymorphism increases the transient outward current (I_to,f_) and abbreviates the action potential duration and the QT interval

**DOI:** 10.1038/s41598-020-67109-z

**Published:** 2020-07-01

**Authors:** David Tinaquero, Teresa Crespo-García, Raquel G. Utrilla, Paloma Nieto-Marín, Andrés González-Guerra, Marcos Rubio-Alarcón, Anabel Cámara-Checa, María Dago, Marcos Matamoros, Marta Pérez-Hernández, María Tamargo, Jorge Cebrián, José Jalife, Juan Tamargo, Juan Antonio Bernal, Ricardo Caballero, Eva Delpón, ITACA Investigators, Joaquín J. Alonso-Martín, Fernando Arribas, Felipe Atienza, Antonio Hernández-Madrid, José Luis López-Sendón, Julián Pérez-Villacastín, Jorge Toquero

**Affiliations:** 1 Department of Pharmacology and Toxicology. School of Medicine. Universidad Complutense de Madrid, Instituto de Investigación Sanitaria Gregorio Marañón. CIBERCV, Madrid, Spain; 2 Centro Nacional de Investigaciones Cardiovasculares (CNIC), Madrid, Spain; 3 Cardiology Department, Hospital General Universitario Gregorio Marañón, Instituto de Investigación Sanitaria Gregorio Marañón. CIBERCV, Madrid, Spain; 4 Department of Internal Medicine/Cardiovascular Medicine, University of Michigan, Ann Arbor, MI USA; 5 Cardiology Department, Hospital Universitario de Getafe, Getafe, Spain; 6 Cardiology Department, Hospital Universitario 12 de Octubre, Madrid, Spain; 7 Cardiology Department, Hospital Universitario Ramón y Cajal, Madrid, Spain; 8 Cardiology Department, Hospital Universitario La Paz, Madrid, Spain; 9 Cardiology Department, Hospital Clínico San Carlos, Madrid, Spain; 10 Cardiology Department, Hospital Universitario Puerta de Hierro, Majadahonda, Spain

**Keywords:** Cardiology, Molecular medicine

## Abstract

Synapse-Associated Protein 97 (SAP97) is an anchoring protein that in cardiomyocytes targets to the membrane and regulates Na^+^ and K^+^ channels. Here we compared the electrophysiological effects of native (WT) and p.P888L SAP97, a common polymorphism. Currents were recorded in cardiomyocytes from mice trans-expressing human WT or p.P888L SAP97 and in Chinese hamster ovary (CHO)-transfected cells. The duration of the action potentials and the QT interval were significantly shorter in p.P888L-SAP97 than in WT-SAP97 mice. Compared to WT, p.P888L SAP97 significantly increased the charge of the Ca-independent transient outward (I_to,f_) current in cardiomyocytes and the charge crossing Kv4.3 channels in CHO cells by slowing Kv4.3 inactivation kinetics. Silencing or inhibiting Ca/calmodulin kinase II (CaMKII) abolished the p.P888L-induced Kv4.3 charge increase, which was also precluded in channels (p.S550A Kv4.3) in which the CaMKII-phosphorylation is prevented. Computational protein-protein docking predicted that p.P888L SAP97 is more likely to form a complex with CaMKII than WT. The Na^+^ current and the current generated by Kv1.5 channels increased similarly in WT-SAP97 and p.P888L-SAP97 cardiomyocytes, while the inward rectifier current increased in WT-SAP97 but not in p.P888L-SAP97 cardiomyocytes. The p.P888L SAP97 polymorphism increases the I_to,f_, a CaMKII-dependent effect that may increase the risk of arrhythmias.

## Introduction

Proper function of cardiac ion channels requires them to be targeted and retained within specific myocyte membrane domains in proximity with specific regulatory molecules^1^. Alterations of ion channel synthesis, membrane trafficking, and/or posttranslational modifications may lead to ion channel function defects that can give rise to acquired or genetically determined arrhythmogenic syndromes^2^. Cardiac Synapse-Associated Protein 97 (SAP97) is an anchoring protein of the MAGUK family encoded by the *DLG1* gene^3^. Its molecule contains different protein-protein interaction domains including three PDZ, one Src-homology-3 (SH3), and one guanylate-kinase (GUK) like domains^4^. SAP97 is one of a number of proteins that target ion channels to specialized domains of the plasma membrane and modulate their activity^4^. Cardiac SAP97 interacts with Kir2.1–2.3 channels responsible for the inward rectifier current (I_K1_)^5,6^ and silencing of SAP97 in cardiomyocytes or in genetically modified mice significantly decreased I_K1_^7,8^. Nav1.5 channels that generate the Na^+^ current (I_Na_) also bind to and co-localize with SAP97 at the intercalated disc^9^. Accordingly, SAP97 silencing decreases I_Na_ in cardiomyocytes and expression systems^6,9^. Kv1.5 channels, underlying the ultra rapid delayed rectifier current (I_Kur_)^10,11^, and Kv4.3/4.2 channels, generating the Ca^2+^-independent transient outward current (I_to,f_), also bind to SAP97^12^. In fact, knocking-down SAP97 in mice significantly reduces both I_Kur_ (named I_K,slow_ in mouse cardiomyocytes) and I_to,f_, prolonging the action potential duration (APD) and the QT interval on the electrocardiogram (ECG)^7^. Accordingly, Gillet *et al*.^7^. proposed that in humans *DLG1* variants might also influence the QT duration by modifying the K^+^ current densities. However, to our knowledge, no data exists linking *DLG1* mutations to ECG abnormalities.

The rs34492126 *DLG1* polymorphism encodes the p.P888L I3-I1A isoform of SAP97 whose total allele frequency is 4.1% (GnomAD, https://gnomad.broadinstitute.org/). p.P888L is the second most frequent of the five most prevalent *DLG1* variants involving a single amino acid change, and is also the most damaging according to several predictive scores (see Supplementary Table I). Previous results obtained when studying the functional consequences of common variants in genes like *SCN5A* or *KCNE1* suggested that they can increase the risk of developing atrial fibrillation (AF)^13,14^.This effect has been attributed to different electrophysiological effects of the polymorphic protein compared with the native (WT) form. Furthermore, common variants might also be genetic modifiers that influence the penetrance and expressivity of Mendelian inherited arrhythmias^15^. Therefore, and considering the role of SAP97 in modulating Na^+^ and K^+^ channels^5,7,9,10,12^, we analyzed the electrophysiological consequences of expressing the common p.P888L SAP97 polymorphism in a cardiac-specific manner in mice generated via intravenous adeno-associated virus (AAV)-mediated gene delivery and in expression systems. Our results demonstrate that compared to the WT form, p.P888L SAP97 significantly shortens the APD and the QT interval as a consequence of a Ca/calmodulin kinase II (CaMKII)-mediated I_to,f_ increase. Our findings strongly suggest that the rs34492126 *DLG1* polymorphism might differentially modify the human cardiac electrophysiological properties.

## Results

We used live, cardiomyocyte-specific expression of human WT (hereinafter referred to as WT-SAP970 or p.P888L-SAP97 in mice via intravenous AAV9-mediated gene delivery as previously described^16^. This method guarantees the robust stability of long-term expression of the gene delivered after a single administration. More importantly, the gene delivery approach obviates the need for complex backcrosses and the maintenance of large colonies of genetically modified animals, resulting in a drastic reduction of the number of animals used^16,17^. Our method yielded a long-lasting (≥24 months) trans-expression, ensuring the homogeneous distribution of the transgene throughout the ventricles (Supplementary Fig. I) and a cardiomyocyte transduction efficiency (quantified by tdTomato immunofluorescence) of >95% (Supplementary Fig. I)^16^. WT-SAP97 and p.P888L-SAP97 trans-expressing mice showed a moderate increase in the expression of the SAP97 protein as demonstrated by WB experiments, being only ~50% greater than the endogenous SAP97 protein in Sham mice (Supplementary Fig. I). Furthermore, WT-SAP97 and p.P888L-SAP97 mice did not show any apparent cardiac or extra-cardiac defects or increased mortality compared with Sham mice.

### p.P888L SAP97 differentially accelerates cardiac repolarization

To test whether expression of p.P888L-SAP97 produces any differential electrophysiological effects *in-vivo* compared with WT-SAP97, we recorded ECGs in the three mouse groups as summarized in Table 1. In anesthetized animals, trans-expression of either WT or p.P888L did not modify the P wave, PR, or RR interval durations, but significantly shortened the QRS (P < 0.01). Importantly, the QT interval in p.P888L-SAP97 mice was significantly shorter than both WT-SAP97 (P < 0.02) and Sham mice.

**Table 1:** ECG parameters measured in mice expressing or not SAP97.

Mice group (N)	P wave (ms)	PR (ms)	QRS (ms)	QT (ms)	RR (ms)
**Sham (N = 4)**	10.1 ± 0.8	45.9 ± 2.7	15.0 ± 0.7	78.2 ± 9.1	139.7 ± 8.2
**WT(N = 9)**	10.8 ± 0.8	41.8 ± 1.9	10.6 ± 0.2*	57.9 ± 3.3*	140.2 ± 8.2
**p.P888L(N = 9)**	10.0 ± 0.4	42.6 ± 1.3	10.8 ± 0.2*	50.6 ± 2.1*^#^	139.4 ± 6.0

Each value represents mean ± SEM of N animals in each group. *P < 0.01 vs SAP97( − ); ^#^P < 0.02 vs SAP97 WT.

**Figure 1:**
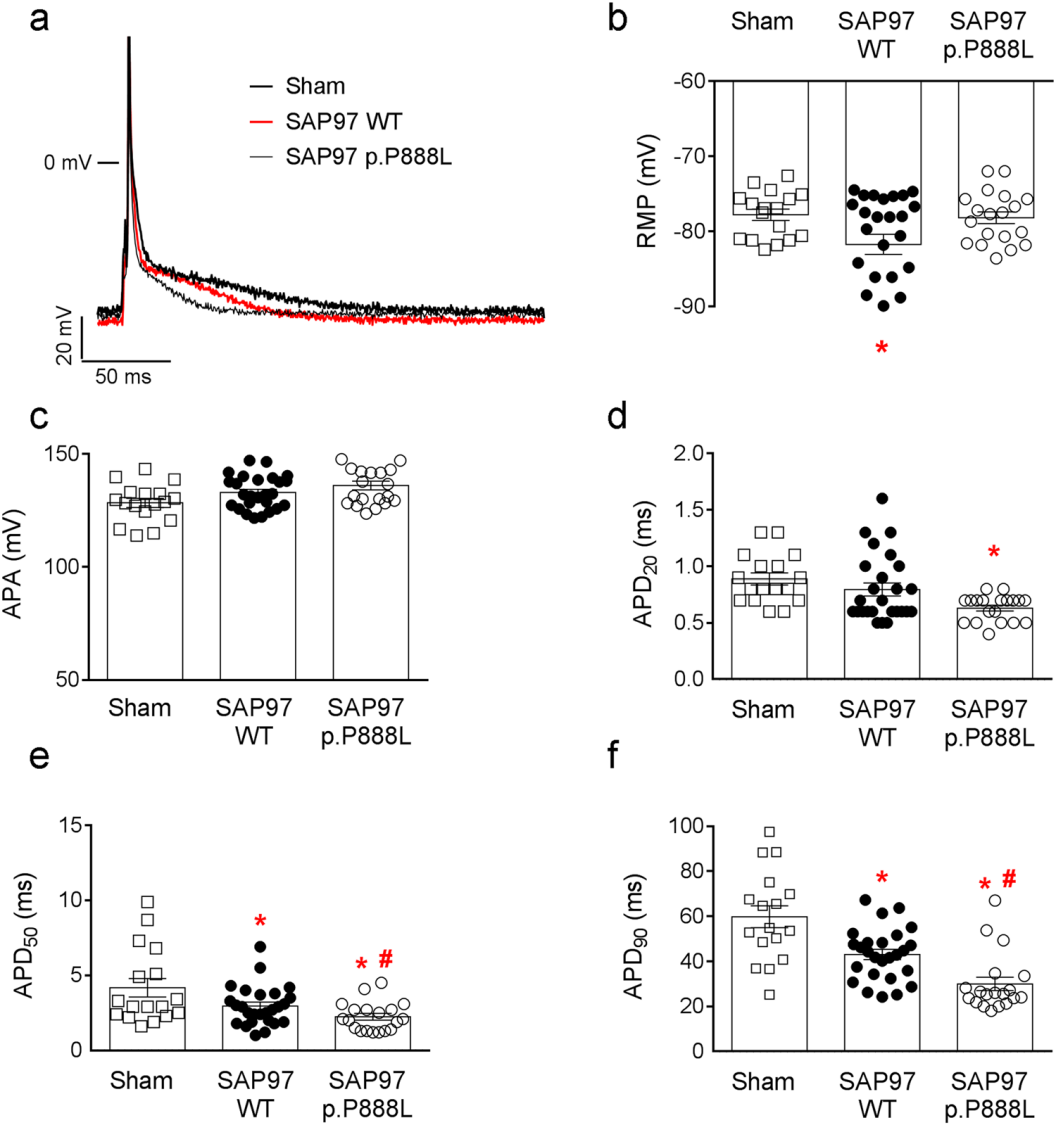
p.P888L SAP97 shortens the APD. (**a**) Superimposed AP recorded in Sham (N = 5 mice), WT-SAP97 (N = 10) and p.P888L-SAP97 (N = 10) cardiomyocytes. (b-f) RMP (**b**), APA (**c**), and APD measured at 20% (**d**), 50% (**e**), and 90% (**f**) of repolarization in cardiomyocytes from the three mouse groups. *P < 0.05 vs Sham. ^#^P < 0.05 vs SAP97 WT.

Next, we recorded APs in cardiomyocytes dissociated from Sham, WT-SAP97 and p.P888L-SAP97 mice (Fig. 1a) to investigate the cellular correlates of the ECG modifications. In WT-SAP97 cardiomyocytes the resting membrane potential (RMP, Fig. 1b) was significantly more hyperpolarized than Sham and p.P888L-SAP97 cardiomyocytes, whereas AP amplitude (APA, Fig. 1c) was similar in all three groups. Of interest, APD measured at 20% repolarization (APD_20_, Fig. 1d) was shorter in p.P888L-SAP97 than WT-SAP97 and Sham cardiomyocytes. On the other hand, APD of WT-SAP97 was significantly shorter than Sham at 50% (APD_50_, Fig. 1e) and 90% (APD_90,_ Fig. 1f) repolarization, and APD of p.P888L-SAP97 was even shorter than WT-SAP97 cardiomyocytes (Fig. 1e–f).

Altogether, the above results demonstrate that *in-vivo* trans-expression of the P888L polymorphism of SAP97 in mice abbreviates the APD, and consequently the QT interval, substantially more than the WT form.

### p.P888L SAP97 increases charge density across Kv4.3 channels

In mouse cardiomyocytes repolarization is mainly due to a rapidly-activating and inactivating transient outward current (I_to,f_), generated by Kv4.3/4.2 channels^18,19^ which is superimposed on a fast-activating slow-inactivating current (I_K,slow_) generated by Kv1.5 channels^20^. Additionally, a slow-activating non-inactivating current (I_ss_) is present. Therefore, I_to,f_ and I_K,slow_ underlie the peak current, while I_K,slow_ and I_ss_ underlie the sustained component. Since Kv4.3/4.2 and Kv1.5 channels are also functionally expressed in human myocardium, we compared the I_to,f_ and I_K,slow_ in the three groups of mice.

**Figure 2:**
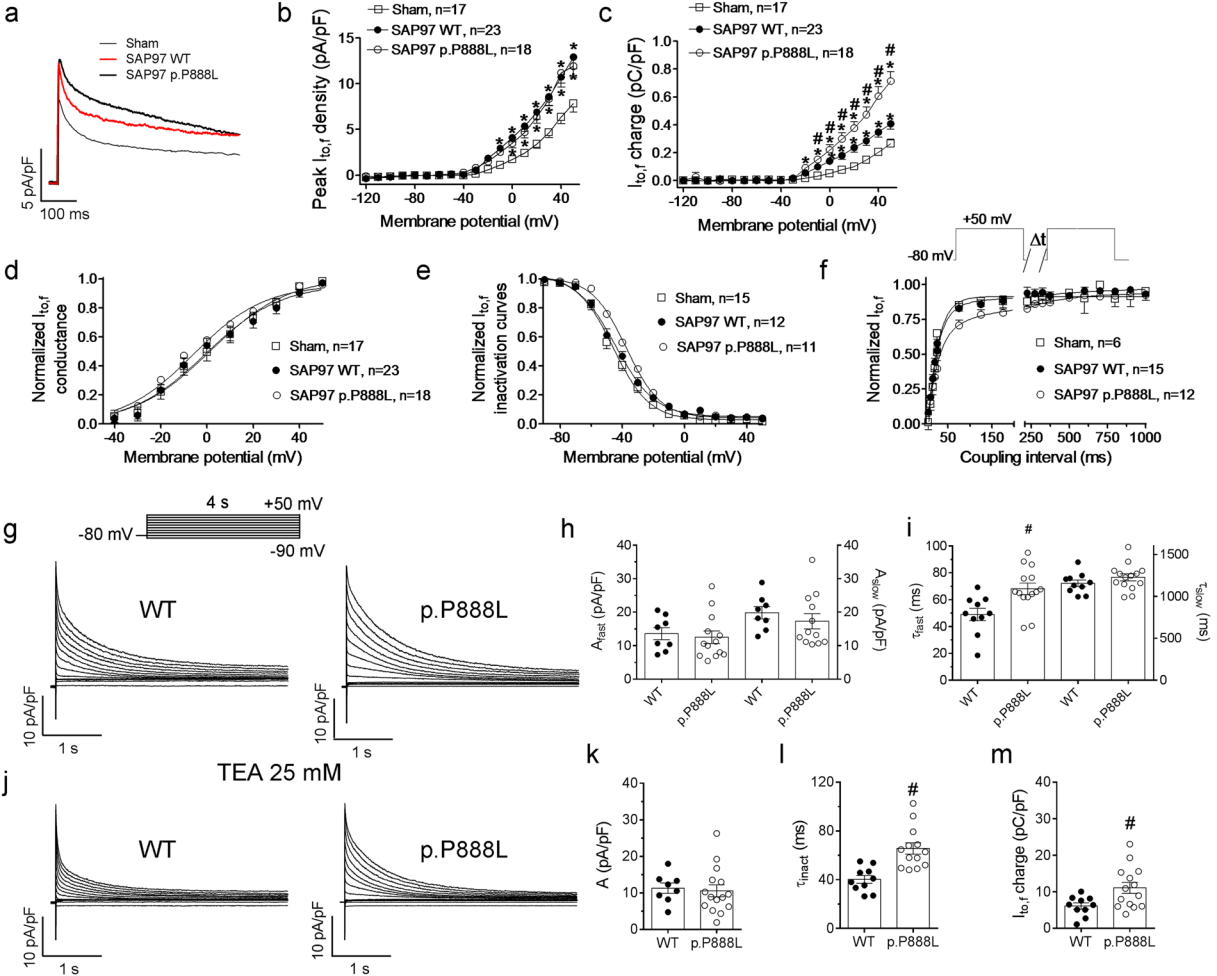
p.P888L SAP97 slows the I_to,f_ inactivation process. (**a**) Outward currents elicited by 500-ms pulses to +50 mV in Sham, WT-SAP97 and p.P888L-SAP97 cardiomyocytes. (b-e) Peak I_to,f_ density-voltage (**b**), I_to,f_ charge density-voltage (**c**), activation (**d**) and inactivation (**e**) curves obtained in cardiomyocytes from the three mouse groups. (**f**) I_to,f_ reactivation kinetics measured by a double-pulse protocol (top). Continuous lines represent the Boltzmann (d-e) and the monoexponential or biexponential fits (f), respectively, to the datapoints. In b-f each point represents the mean ± SEM of “n” experiments/cardiomyocytes dissociated from five mice of each group. (**g**) K^+^ current traces elicited by 4-s pulses in WT-SAP97 and p.P888L-SAP97 cardiomyocytes. (**h**) Density of the fast and slow components derived from the biexponential fit to the decline of the currents elicited by 4-s pulses to +50 mV in WT-SAP97 and p.P888L-SAP97 cardiomyocytes. (**i**) Fast and slow time constants of inactivation obtained by fitting a biexponential function to the K^+^ current traces elicited by 4-s pulses in WT-SAP97 and p.P888L-SAP97 cardiomyocytes. (**j**) K^+^ current traces elicited by 4-s pulses in WT-SAP97 and p.P888L-SAP97 cardiomyocytes in the presence of 25 mM TEA. (**k**) Density of the fast component derived from the monoexponential fit to the decline of the currents elicited by 4-s pulses to +50 mV in WT-SAP97 and p.P888L-SAP97 cardiomyocytes in the presence of 25 mM TEA. (**l**) Inactivation time constants obtained by fitting a monoexponential function to the K^+^ current traces elicited by 4-s pulses in WT-SAP97 and p.P888L-SAP97 cardiomyocytes in the presence of 25 mM TEA. (**m**) I_to,f_ charge-density generated by 4-s pulses to +50 mV in WT-SAP97 and p.P888L-SAP97 cardiomyocytes in the presence of 25 mM TEA. *P < 0.05 vs Sham, ^#^P < 0.05 vs SAP97 WT.

First, we measured I_to,f_ amplitude as the difference between the peak and the steady-state current at the end of short (500-ms) pulses^21^. Trans-expression of either form of SAP97 significantly and similarly increased the peak-I_to,f_ density (Fig. 2a,b, P < 0.05). The effects of SAP97 on the peak I_to,f_ are expected and may be attributed to the clustering of functional Kv4.3 channel into the plasma membrane^12^. To quantify the amount of K^+^ crossing the membrane through Kv4.3/Kv4.2 channels (i.e., I_to,f_ charge) we integrated the peak-I_to,f_ traces to calculate the area. Figure 2c demonstrates that in cardiomyocytes from both WT-SAP97 and p.P888L-SAP97 mice the I_to,f_ charge-density was significantly greater. More important, the I_to,f_ charge-density of p.P888L-SAP97 cardiomyocytes was almost twice that of WT-SAP97 cardiomyocytes.

The voltage-dependence of I_to,f_ activation and inactivation in WT-SAP97 and Sham cells were undistinguishable, while they were significantly hyperpolarized and depolarized, respectively, in p.P888L-SAP97 myocytes (Fig. 2d–e, Supplementary Table II). Furthermore, in cardiomyocytes from p.P888L-SAP97 mice the I_to,f_ reactivation was significantly slowed by the presence of a new slow component (Fig. 2f, Supplementary Table II). Thus, except for the reactivation process, it seems that p.P888L SAP97 produced a gain-of-function-like effect on I_to,f_.

Figure 2g shows current traces generated by 4-s depolarizing pulses in WT and p.P888L cardiomyocytes. Following previously described methods^19^, we fitted biexponential functions to the decay of the currents and considered the fast and slow components of inactivation as I_to,f_ and I_K,slow_, respectively. The amplitude of the fast component (A_fast_) was similar in WT-SAP97 and p.P888L-SAP97 cardiomyocytes (Fig. 2h), indicating that the peak-I_to,f_ density was not different in both groups of mice. However, the decay of this component was significantly slower in p.P888L-SAP97 myocytes (Fig. 2i, Supplementary Table II). Regarding the slow component, the amplitude at +50 mV (A_slow_) (Fig. 2h) and the τ of decay (Fig. 2i) were similar in WT-SAP97 and p.P888L-SAP97 cardiomyocytes (Supplementary Table II). These results suggested that the I_K,slow_ was similarly affected by the presence of WT and p.P888L SAP97.

Superfusion of 25 mM tetraethylammonium (TEA) reduces by 60% both the I_K,slow_ and the I_ss_, leaving the I_to,f_ almost unaffected^19^. Therefore, the inactivating component of the TEA-resistant current can be assimilated to the I_to,f_ (Fig. 2j). We fitted the decay of the TEA-resistant current with a single exponential whose amplitude was superimposable on the A_fast_ in the absence of TEA and similar between WT-SAP97 and p.P888L-SAP97 cardiomyocytes (Fig. 2k). Conversely, the current decay was significantly slower in p.P888L than in WT SAP97 cardiomyocytes (Fig. 2j,l, Supplementary Table II). Consequently, the charge density was significantly greater in p.P888L-SAP97 cardiomyocytes (Fig. 2m).

**Figure 3:**
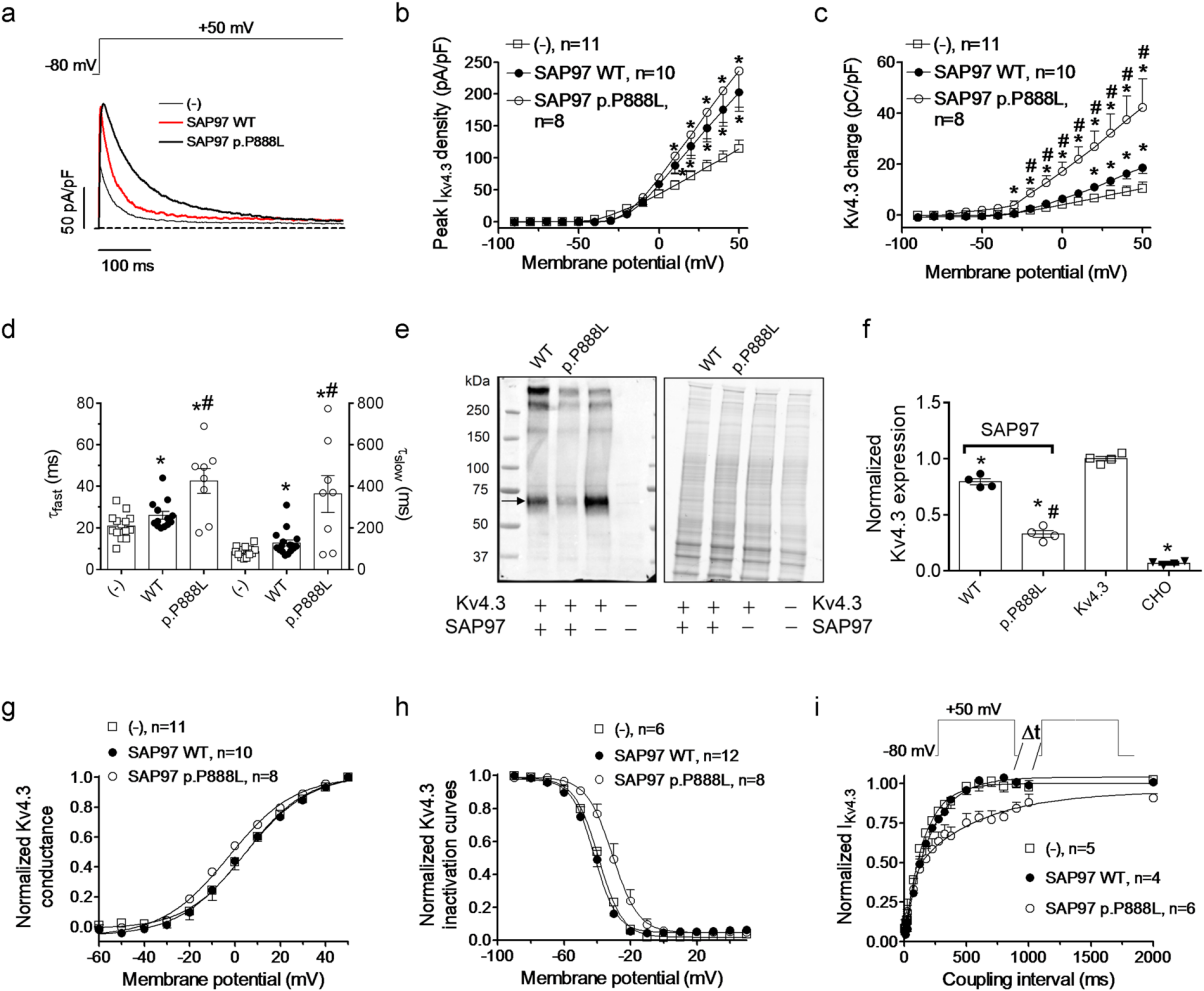
Time- and voltage-dependent effects of WT and p.P888L SAP97 on I_Kv4.3_. (**a**) Representative I_Kv4.3_ traces recorded by applying 500-ms pulses from −80 to +50 mV in CHO cells expressing Kv4.3 channels co-transfected or not with WT and p.P888L SAP97. (b and c) Mean peak I_Kv4.3_ density (**b**) and Kv4.3 charge-density (**c**) measured in the 3 experimental groups. (**d**) Fast and slow time constants of inactivation obtained by fitting a biexponential function to I_Kv4.3_ recorded by applying pulses from −80 to +50 mV in the 3 experimental groups. (**e**) Western Blot image (left) and its corresponding stain-free gel (right) showing Kv4.3 expression in CHO cells transfected or not (right lane) with Kv4.3 channels in the absence (third lane) or in the presence of WT or p.P888L SAP97. (**f**) Mean densitometric analysis of Kv4.3 levels normalized to total protein. (**g** and **h**) Conductance-voltage (**g**) and inactivation (**h**) curves for I_Kv4.3_ recorded in the 3 experimental groups. (**i**) Time course of I_Kv4.3_ recovery measured by a double-pulse protocol (top) in the 3 experimental groups. Continuous lines in panels g and h represent the fit of Boltzmann functions to the data. Continuous lines in panel i represent the monoexponential or biexponential fit to the data. Each point represents the mean ± SEM of “n” experiments/cells. *P < 0.05 vs (−). ^#^P < 0.05 vs SAP97 WT.

To better dissect the effects of both SAP97 forms on I_to,f_ and I_K,slow_, we conducted experiments in transfected cells expressing Kv4.3 and hKv1.5 channels, respectively, in the presence and the absence of either WT or p.P888L SAP97. In Fig. 3, SAP97 WT significantly increased peak Kv4.3 currents (I_Kv4.3_) (panels a-b), slowed the inactivation kinetics (panel d), and augmented the charge crossing the membrane through Kv4.3 channels (panel c) transiently expressed in Chinese hamster ovary cells (CHO). p.P888L SAP97 also increased peak I_Kv4.3_, but slowed the inactivation kinetics. Therefore, p.P888L SAP97 augmented the I_Kv4.3_ charge density much more than WT SAP97 (P < 0.05 vs WT SAP97) (Fig. 3a–d). Furthermore, p.P888L, but not WT SAP97, significantly hyperpolarized and depolarized the activation and inactivation curves, respectively (Fig. 3g,h), and significantly slowed the I_Kv4.3_ reactivation kinetics (Fig. 3i) (Supplementary Table II). Hence, the differential effects of WT vs p.P888L SAP97 on I_Kv4.3_ in CHO cells were quantitatively and qualitatively similar to those shown above for I_to,f_ in mouse cardiomyocytes. As demonstrated by the WB experiments in Fig. 3e,f, another important difference between the two SAP97 forms is that WT slightly but significantly decreased the total amount of Kv4.3 protein in CHO cells, while, unexpectedly, p.P888L markedly decreased the total Kv4.3 expression (Fig. 3e,f).

We recorded currents generated by hKv1.5 channels (I_Kv1.5_) in *Ltk*^*-*^ cells stably expressing WT or p.P888L SAP97 and compared them with untransfected cells. Supplementary Fig. II confirmed that WT and p.P888L SAP97 similarly increased the I_Kv1.5_ at potentials positive to +10 mV and that none produced any voltage- or time-dependent modification of the current (Supplementary Table II). We attribute the I_Kv1.5_ increasing effects of SAP97 native and polymorphic forms to clustering of Kv1.5 channels in the cardiomyocyte membrane^10^.

### Mechanistic insight

**Figure 4:**
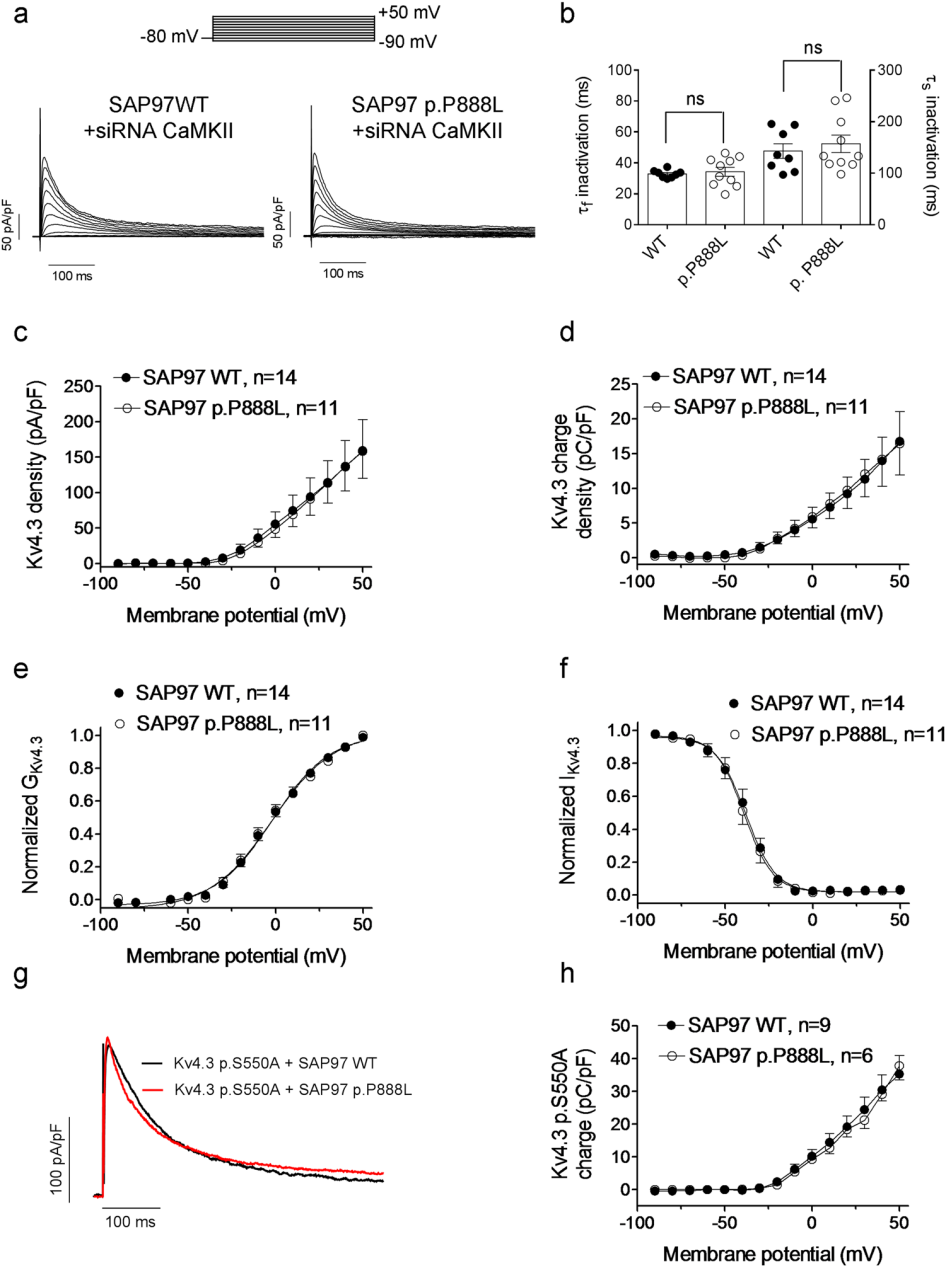
The effects of p.P888L SAP97 on Kv4.3 channels are CaMKII-dependent. (**a**) I_Kv4.3_ traces recorded in CaMKII-silenced CHO cells co-transfected with WT or p.P888L SAP97. (**b**) τ_f_ and τ_s_ of I_Kv4.3_ inactivation obtained from the biexponential fit of the decline of the I_Kv4.3_ recorded at +50 mV in CaMKII-silenced cells. (**c-f**) Peak I_Kv4.3_ density-voltage (**c**), I_Kv4.3_ charge density-voltage (**d**), and activation (**e**) and inactivation (**f**) curves obtained in CaMKII-silenced CHO cells co-transfected with WT or p.P888L SAP97. (**g**,**h**) Traces (**g**) and charge density-voltage relationships (**h**) generated by p.S550A Kv4.3 channels co-expressed with WT or p.P888L SAP97. In c-f and h each point represents the mean ± SEM of “n” experiments/cells of at least 3 different dishes.

CaMKII inhibition accelerates inactivation of I_to,f_ in cardiomyocytes and I_Kv4.3_ in heterologous expression systems^22,23^. Conversely, CaMKII does not have any effect on the Kv4.2 inactivation kinetics under basal conditions in either rat cardiomyocytes or expression systems^24,25^. Further, Kv4.3, SAP97, and CaMKII form a tripartite complex that allows the regulation of Kv4.3 channels by CaMKII^12^. Therefore, we decided to specifically analyze whether the differential effects of p.P888L SAP97 on Kv4.3 channels can be attributed to CaMKII by silencing its expression using siRNAs^6,26^.Transfection of CHO cells with a combination of siRNAs against all CaMKII isoforms reduced their expression by ~55% (Supplementary Fig. III). In CaMKII-silenced cells, the I_Kv4.3_ inactivation kinetics (and consequently the τ_fast_ and τ_slow_) was similar in cells expressing WT or p.P888L SAP97 (Fig. 4a,b). Accordingly, the charge-density voltage relationships obtained in CaMKII-silenced cells expressing WT or p.P888L SAP97 overlapped (Fig. 4d). Furthermore, the peak I_Kv4.3_ density (Fig. 4c) and the voltage dependence of Kv4.3 activation (Fig. 4e) and inactivation (Fig. 4f) were undistinguishable in CaMKII-silenced cells expressing WT or p.P888L SAP97 (Supplementary Table II).

Additionally, we tested the CaMKII involvement by inhibiting the enzyme (consequently Fig. III). In cells co-transfected with p.P888L SAP97, CaMKII inhibition with KN93^27,28^ significantly accelerated I_Kv4.3_ inactivation (Supplementary Table II) and decreased the charge-density in cells transfected with either WT or p.P888L SAP97 (Supplementary Fig. III). However, the decrease in I_Kv4.3_ charge density was greater in p.P888L than WT SAP97 cells. In fact, at +50 mV the I_Kv4.3_ charge-density reduction was 51.0% and 33.7% in the presence of p.P888L and WT SAP97, respectively (P < 0.05). Similar effects were obtained in cells dialyzed with autocamtide-2-related inhibitory peptide (AIP), another CaMKII inhibitor (Supplementary Fig. III) (n = 5, P < 0.05)^22^.

**Figure 5:**
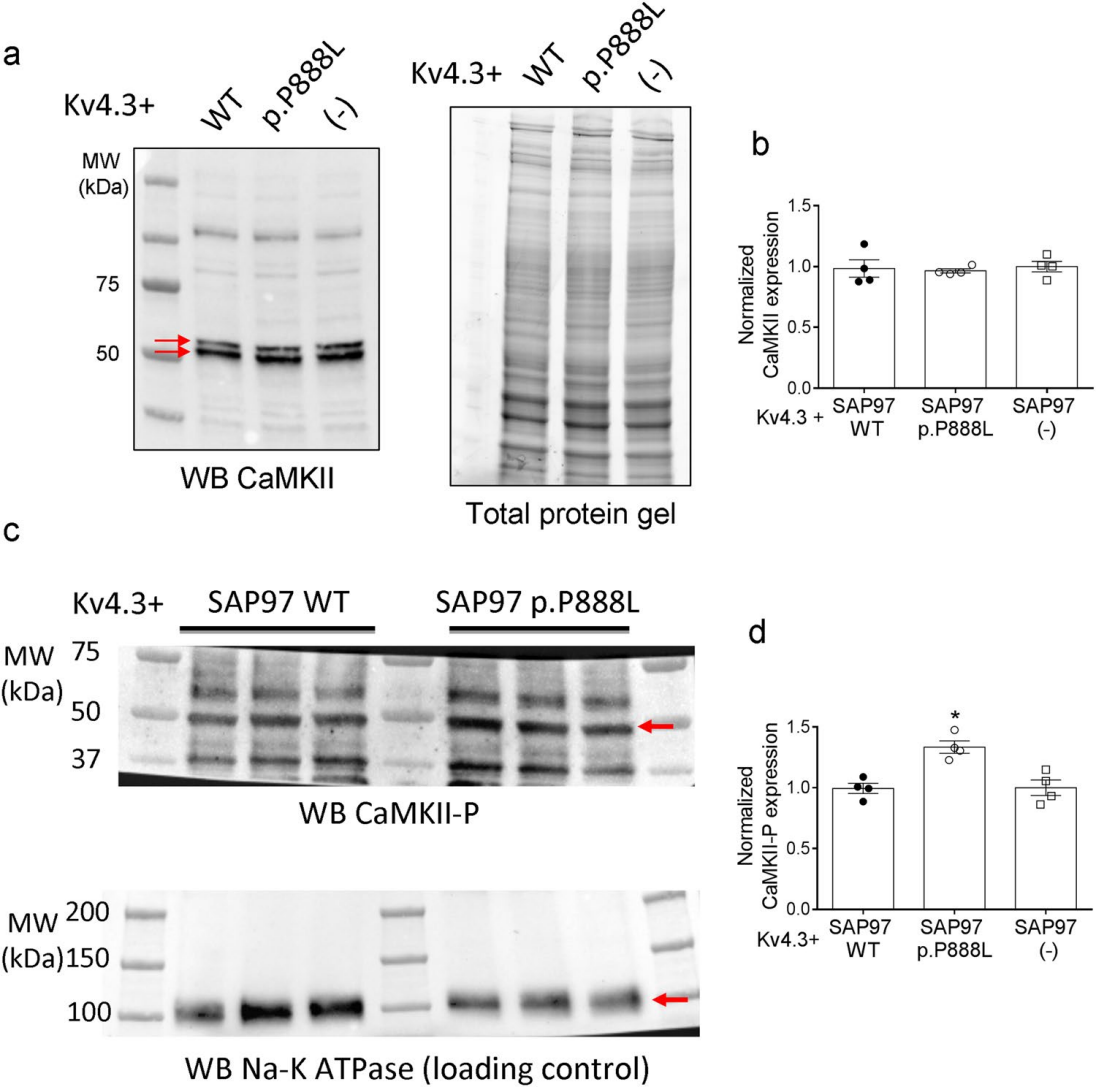
CaMKII and phosphorylated-CaMKII expression in cells co-transfected with WT or p.P888L SAP97. (**a**) WB images and their corresponding stain-free gels showing CaMKII expression (arrows) in CHO cells transfected with Kv4.3 channels alone or in the presence of WT or p.P888L SAP97. (**b**) Mean densitometric analysis of CaMKII levels normalized to total protein (n = 4). (**c**) (Top) WB images of phosphorylated-CaMKII expression in CHO cells co-transfected with Kv4.3 channels and WT or p.P888L SAP97. Na^+^-K^+^ ATPase was used as a loading control (bottom). (**d**) Mean densitometric analysis of phosphorylated-CaMKII levels normalized to the Na^+^-K^+^ ATPase density (n = 4). *P < 0.05 vs Kv4.3 and WT SAP97.

In rat Kv4.3 channels the primary site for CaMKII phosphorylation is Ser at position 550^23^. Figure 4g shows I_Kv4.3_ traces and Fig. 4h the charge-density curves for p.S550A Kv4.3 channels when co-expressed with WT or p.P888L SAP97. Figure 4h demonstrates that compared with WT, p.P888L SAP97 failed to increase the charge density of p.S550A Kv4.3 channels (P > 0.05). WB analysis in CHO cells expressing Kv4.3 channels demonstrated that the total expression of CaMKII was independent of the SAP97 form expressed (Fig. 5a,b). However, p.P888L SAP97 slightly but significantly increased the phosphorylated-(activated) form of CaMKII (Fig. 5c,d) (P < 0.05). Altogether, these results demonstrate that p.P888L SAP97 increases I_to,f_ by significantly augmenting CaMKII effects on Kv4.3 channels.

### Protein-protein docking between CaMKIIδ and SH3-GUK is stronger for p.P888L than WT

**Figure 6:**
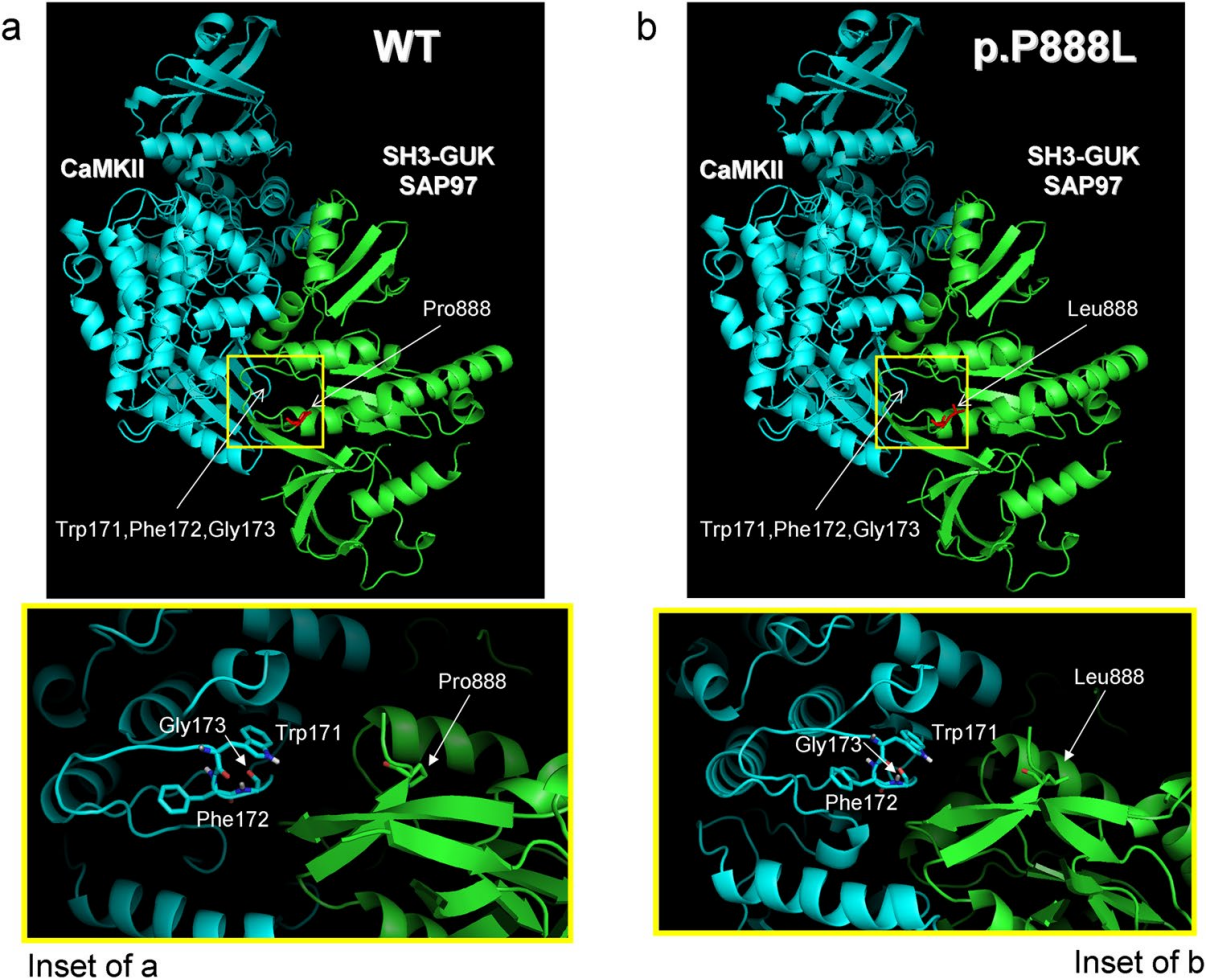
Protein-protein docking between CaMKIIδ and SH3-GUK domain of SAP97. (**a** and **b**) Representative images summarizing the results of the blind docking study between CaMKIIδ (blue) and WT (Panel a) or p.P888L (Panel b) forms of the SH3-GUK domain of SAP97 (green) conducted by using the ClusPro web server. The images that correspond to the center (i.e. the structure that has the highest number of neighbour structures in the cluster) of the most populated cluster (#0 in Table S2) for each condition (WT or p.P888L) are shown. Pro-888 (Panel a) and Leu-888 (Panel b) of SAP97 have been coloured in red. The location of Trp-171, Phe-172, and Gly-173 of CaMKIIδ are indicated by the arrows. The insets in Panels a and b show the region surrounding Pro-888 (**a**) and Leu-888 (**b**) of SAP97 (rectangles) in an expanded scale indicating the specific location of Trp-171, Phe-172, and Gly-173 of CaMKIIδ.

It has been suggested that the presence of the Leu residue at position 888 induces a SAP97 conformational change that favours direct CaMKII binding^29^. This would explain the slight but significant increase in phosphorylated-(activated) CaMKII observed in cells transfected with p.P888L SAP97. Therefore to gain further insight and identify putative differences in the binding of CaMKIIδ to the SH3-GUK region of WT and p.P888L SAP97^30,31^, we conducted protein-protein docking experiments on the ClusPro web server^30^. We used the crystallized structures of the SH3-GUK domain spanning between SAP97 residues 582 and 908 (including the I3 and I5 inserts) and of CaMKIIδ that includes residues 8 to 300. In the SH3-GUK domain of SAP97, the proline that is equivalent to Pro-888 is located at position 906. The Pro-to-Leu substitution at this position yielded the mutated form of the SH3-GUK domain of SAP97 (named as p.P888L). Analysis using ClusPro web server yields results categorized in 30 clusters that are ranked based on cluster size (number of conformations/members in the cluster). The docking results of the first 5 clusters in the presence of WT or p.P888L SH3-GUK domain of SAP97 are summarized in Supplementary Table III, while Fig. 6 displays the results of the most favorable conformation (center) of the most populated cluster (#0) in each condition. As shown in Fig. 6a, the Pro-888 is located at the end of the GUK domain and forms part of an antiparallel β sheet with the fifth β strand of the SH3 domain, suggesting that it may participate in the interaction between both domains. The SH3-GUK interaction has been proposed to have important functional implications and, indeed, it is considered that both domains form an integral structural unit^31^. The preliminary results of the docking analysis suggest that Pro-888 is in the vicinity of a CaMKIIδ region comprising Trp-171, Phe-172, and Gly-173 (inset in Panel a). Interestingly, Pro to-Leu substitution provokes a subtle conformational change in the SH3-GUK domain of SAP97 (Panel b) leading to a different docking with CaMKIIδ (inset in Panel b). Importantly, the Pro-to-Leu mutation-induced conformational change would increase the number of conformations in 3 out 5 of the most populated clusters (Supplementary Table III), increasing from 50 to 55 conformations in the cluster #0. Therefore, considering that in this docking model the size of a cluster is approximately proportional to its probability^30^, the results obtained suggest that the presence of Leu-888 would allow the SH3-GUK domain of SAP97 to get a more favorable conformation for binding to CaMKIIδ.

### p.P888L SAP97 and the channels underlying I_Na_ and I_K1_

**Figure 7:**
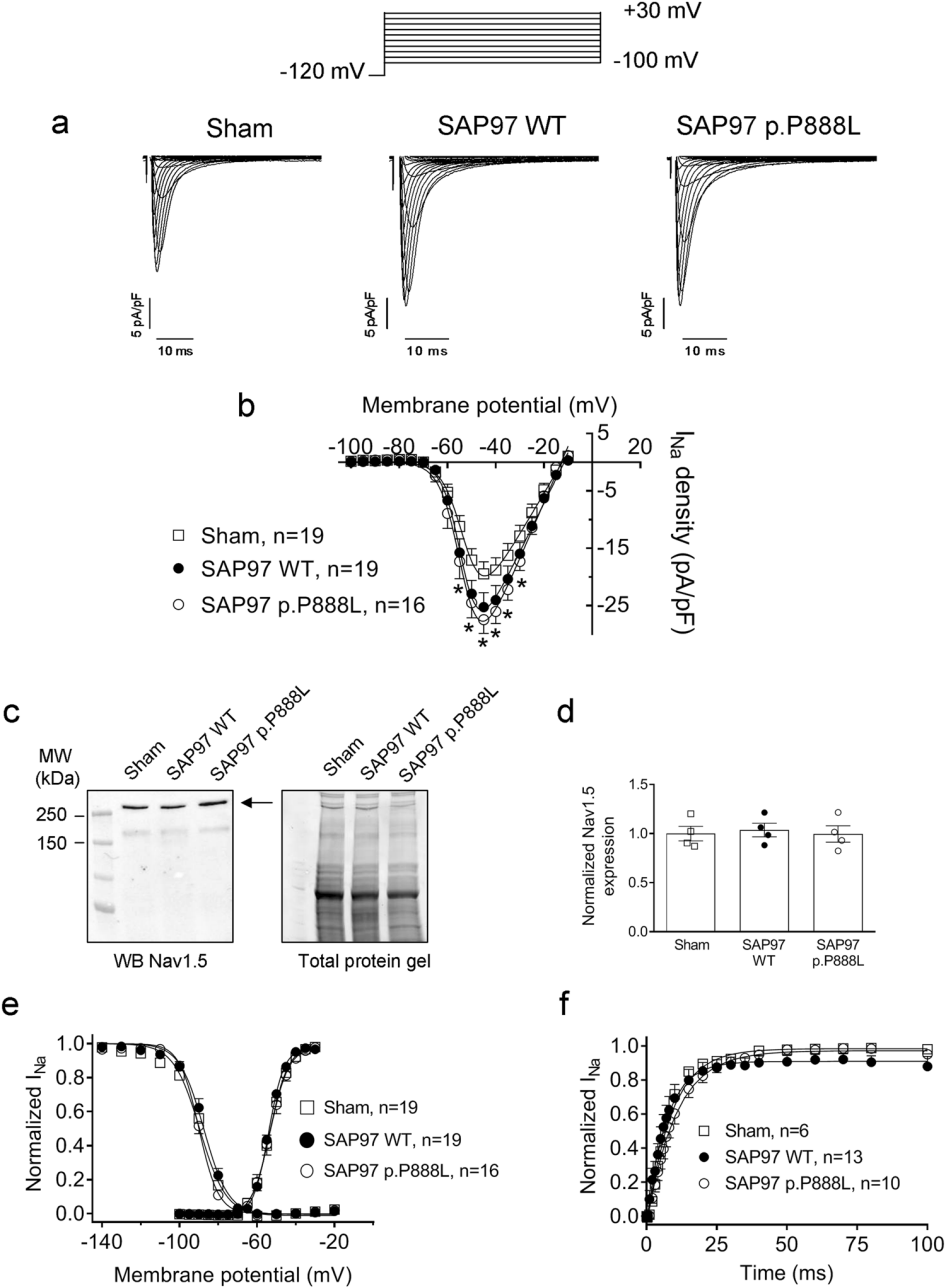
WT and p.P888L SAP97 similarly increased I_Na_ density. (**a**,**b**) Traces (**a**) and density-voltage relationships (**b**) of the I_Na_ recorded in Sham, WT and p.P888L SAP97 mouse cardiomyocytes. (**c**) WB image and its corresponding stain-free gel showing Nav1.5 (arrow) expression in ventricular tissue from Sham, WT and p.P888L SAP97 mice. (**d**) Mean densitometric analysis of Nav1.5 levels normalized to the total protein. (**e**) Inactivation and activation curves for I_Na_ recorded in cardiomyocytes from the three mouse groups. Continuous lines represent the Boltzmann fit of datapoints. (**f**) Time course of I_Na_ reactivation measured in cardiomyocytes from the three mouse groups. In b, e and f each point represents the mean ± SEM of “n” experiments/cardiomyocytes from five mice of each group. *P < 0.05 vs Sham mice.

Finally, we also tested the putative differential effects of p.P888L SAP97 over I_Na_ and I_K1_ since SAP97 interacts with the channels underlying both currents^6–9,32^. Figure 7a,b demonstrate that trans-expression of either WT or p.P888L SAP97 similarly increased the density of the I_Na_ peak (n ≥ 16, P > 0.05). Neither WT nor p.P888L SAP97 modified the inactivation kinetics of peak currents (Supplementary Table IV). The I_Na_ increasing effects of WT and p.P888L SAP97 were not associated with an augmentation of the total Nav1.5 channel expression, as suggested by WB experiments in samples from the ventricles of each of the three mouse groups (Fig. 7c,d). Voltage dependence of the I_Na_ activation and inactivation was also analyzed. Figure 7e and Supplementary Table IV demonstrate that trans-expression of WT and p.P888L SAP97 did not modify the midpoint or the slope of the activation and inactivation curves (n ≥ 16, P > 0.05). Finally, Fig. 7f shows the time course of the I_Na_ reactivation in the three groups of cardiomyocytes demonstrating an almost overlap of the monoexponential curves fitting the data. In fact, the time constants of I_Na_ reactivation kinetics were not modified by either WT or p.P888L SAP97 (n ≥ 6, P > 0.05) (Supplementary Table IV).

**Figure 8:**
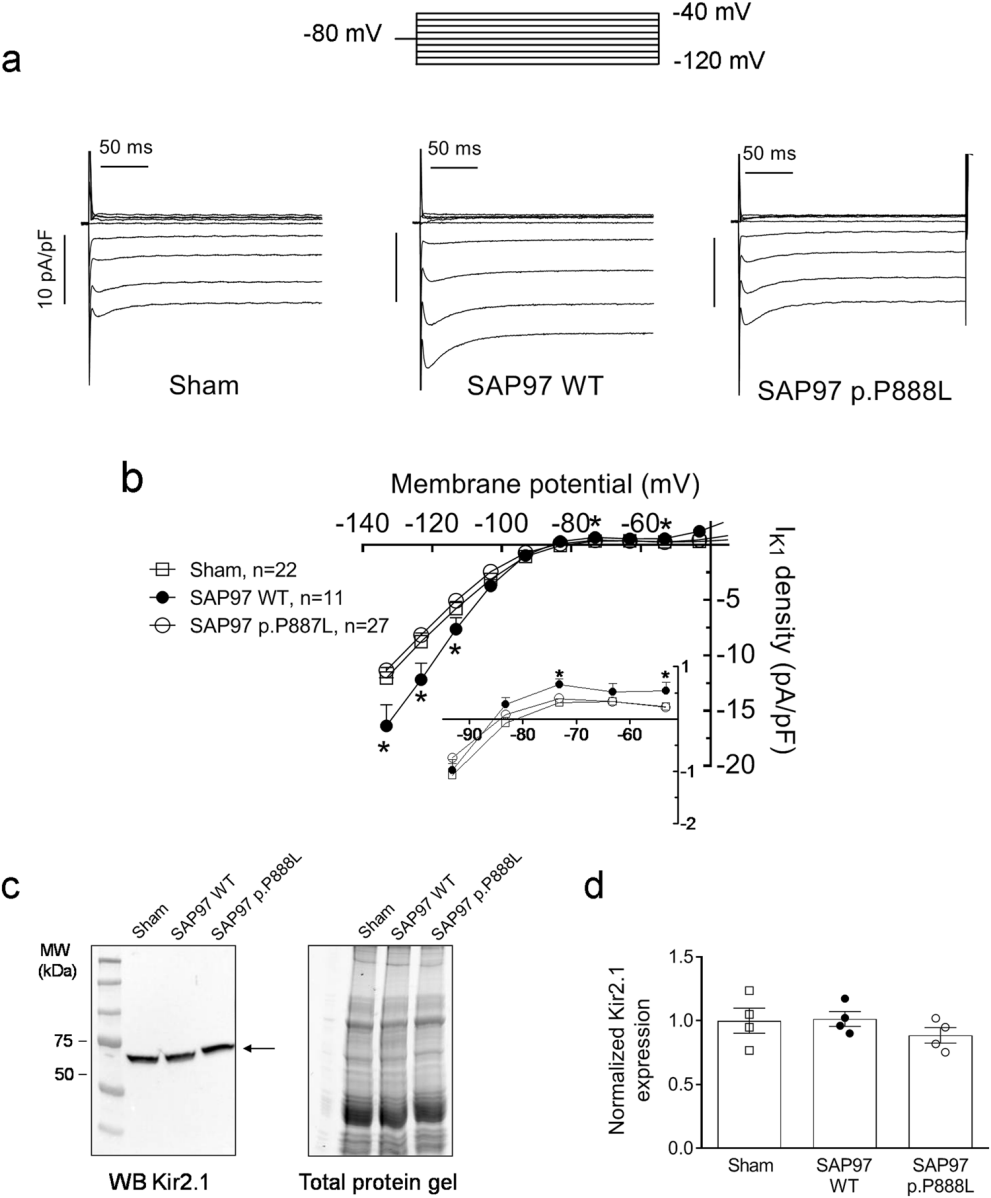
WT, but not p.P888L, SAP97 increases I_K1_ density. (**a**,**b**) Traces (**a**) and current density-voltage curves (**b**) of I_K1_ recorded in Sham, WT and p.P888L SAP97 cardiomyocytes. The inset in b represents data in an expanded scale. Each point represents the mean ± SEM of “n” experiments/cardiomyocytes from five mice of each group. *P < 0.05 vs Sham mice. (**c**) WB image and its corresponding stain-free gel showing Kir2.1 (arrow) expression in ventricular tissue from the three mouse groups. (**d**) Mean densitometric analysis of Kir2.1 levels normalized to the total protein.

In Fig. 8a, I_K1_ also increased in WT SAP97 trans-expressing mice, an effect that was evident at voltages that were both negative and positive (physiologically relevant) to the K^+^ reversal potential (Fig. 8b). Conversely, p.P888L SAP97 failed to increase I_K1_ at any of the membrane potentials tested (Fig. 8a,b). In WB experiments neither WT nor p.P888L SAP97 modified the total amount of Kir2.1 protein in myocardial samples from each of the mouse groups (Fig. 8c,d).

## Discussion

Here we provide the first demonstration that the cardiac-specific expression of the p.P888L SAP97 polymorphism in mice *in vivo* produces a CaMKII-dependent increase of the I_to,f_ charge that leads to shortening of the APD and the QT interval. Conversely, p.P888L and WT SAP97 produce almost identical increasing effects on I_K,slow_ and I_Na_, while they differently modify I_K1_.

Our results demonstrate that trans-expression of human WT SAP97 increased the I_to,f_, I_K,slow_, I_K1_ and I_Na_ in mouse cardiomyocytes. Therefore, the RMP was slightly hyperpolarized and AP repolarization was accelerated. These results are diametrically opposite to those reported by Gillet *et al*. in SAP97-knockout mice showing reduced I_to,f_, I_K,slow_, and I_K1_, and APD prolongation, except for I_Na_, which was not modified in SAP97-knockout mice^7^. As such, our results agree with previous data^5,9,10,12^ adding further support to the contention that SAP97 is a scaffolding protein that functions forming complexes that anchor diverse channels to the plasma membrane, an effect that contributes to generate robust ionic currents^3,4^.

The p.P888L polymorphism is the second most frequent among the *DLG1* variants that involve an amino acid change. Furthermore, according to several predictive scores (Supplementary Table I) this *DLG1* variation is the most damaging among the five most frequent. To the best of our knowledge, the observation that, compared with WT, p.P888L SAP97 markedly increases the I_to,f_ and I_Kv4.3_ charge by slowing the inactivation kinetics of Kv4.3 channels is novel. Furthermore, demonstrating that p.P888L SAP97 shifted the Kv4.3 activation and inactivation curves toward more negative and positive potentials, respectively, is also original. This gain-of-function-like effect produced by p.P888L is quite similar to that produced by CaMKII-mediated phosphorylation of Kv4.3 channels. p.P888L SAP97 also slowed Kv4.3 recovery from inactivation. Regarding the effects of CaMKII on the recovery from inactivation of Kv4.3/I_to,f_, there are discrepancies. Indeed, different groups reported a CaMKII-induced acceleration, slowing, and also no effect^22,23,33^. Under our experimental conditions, it seems that CaMKII slowed Kv4.3 recovery from inactivation in agreement with the results obtained by Groen and Bähring^33^. Furthermore, the effects produced by p.P888L SAP97 on Kv4.3 channel expressed in CHO cells (in which KChIP2 was not co-expressed) were quite similar to those observed in mouse cardiomyocytes. This further supports the contention that the polymorphism does not act by modifying the modulatory role of the β-subunits associated with Kv4.3 channels, including KChIPs. Fundamentally, our results strongly suggest that p.P888L SAP97 increases the I_to,f_ charge via a CaMKII-dependent mechanism that leads to phosphorylation of the S550 residue of the Kv4.3 channel, an effect that is abolished by silencing or inhibiting the enzyme.

In the mouse ventricles, I_to,f_ is generated by both Kv4.3 and 4.2 channels^20^. Since CaMKII does not have any effect on Kv4.2 inactivation kinetics under basal conditions either in rat cardiomyocytes or expression systems^24,25^, it seems reasonable to assume that the slowing of I_to,f_ inactivation in p.P888L-SAP97 cardiomyocytes is due to CaMKII-dependent phosphorylation of Kv4.3, but not of Kv4.2 subunits.

Previous reports demonstrated that in mouse cardiomyocytes, overexpression of CaMKIIδ (the predominant cardiac isoform) significantly decreased the Kv4.3 channel expression and the peak-I_to,f_ density^28^. We demonstrate that transfection with p.P888L SAP97 increases the level of phosphorylated-(activated) CaMKII, which somehow markedly reduces the expression of Kv4.3 channels. Yet the increase in phosphorylated-CaMKII was not accompanied by decrease in peak-I_to,f_. The apparent paradox may be explained, at least in part, by the ability of oligomerized SAP97 to cluster ion channels (including Kv4.3 ones) at the cardiomyocyte membrane what increases the current density generated by those channels^4,12^. Thus, we hypothesize that the peak-I_to,f_ decreasing effects of CaMKII activation are overcome by the SAP97-mediated clustering of Kv4.3 channels at the membrane.

Kv4.3 channels competitively bind to CaMKII at the calmodulin binding site^34^. This binding is independent of the auto-phosphorylation status of CaMKII^34^. Additionally, Kv4.3 interacts, by means of its C-terminal PDZ-binding domain (SAL), with the PDZ domains of SAP97 leading to the formation of a tripartite complex with CaMKII^12^. Furthermore, phosphorylated-CaMKII can interact directly with SAP97 isoforms provided they exhibit the I3-I5 domains in their C-terminal region^29^. The cardiac SAP97 isoforms exhibit the I3-I5 inserts, and the P888 residue is near the CaMKII binding site in this region. Therefore, to determine whether the p.P888L mutation favours a more robust CaMKII binding^29^, we conducted numerical protein-protein docking experiments. Our initial tests using the ClusPro web server, which is a widely used tool for protein-protein docking^30^, predict that p.P888L increases the cluster size and makes the likelihood of forming a CaMKIIδ-p.P888L complex higher than a CaMKIIδ-WT complex. Augmented binding of activated-CaMKII to p.P888L SAP97 would explain the further increase in the charge crossing Kv4.3 channels observed in the presence of the polymorphism.

Our results demonstrate that the p.P888L SAP97 polymorphism increases I_K,slow_ and I_Na_. Thus, we surmise that p.P888L also anchors Kv1.5 and Nav1.5 channels similarly as WT SAP97 does. Conversely, p.P888L SAP97 was unable to increase I_K1_ and, in fact, it did not hyperpolarize the RMP in mouse cardiomyocytes. However, this effect seems to have negligible consequences at least at the ECG level. Finally, it is worth mentioning that both forms of SAP97 shortened the QRS interval, probably because of the I_Na_ increase.

APD and the QT interval are significantly shorter in p.P888L-SAP97 than WT-SAP97 mice. Unfortunately, large differences among currents responsible for the cardiac repolarization in humans and mice limit the extrapolation of the results^20^. However, in humans, I_to,f_ is generated by Kv4.3 channels and is also regulated by CaMKII^22^. Thus, it can be hypothesized that in subjects carrying the p.P888L SAP97 polymorphism the cardiac I_to,f_ charge is greater than in those expressing the WT form. The relative importance of the I_to,f_ in shaping the height and the duration of the plateau phase and the repolarization of the APs in the human myocardium is variable among the different tissues^21,35^. I_to,f_ density is greater in the atria than in the ventricles, and also greater in epicardial than in endocardial ventricular layers^21,35^.Thus, the duration of the atrial and epicardial AP may be shorter in subjects carrying the polymorphism than in WT carriers. In fact, it has been recently demonstrated that in humans the slowing of I_to,f_ inactivation results in dramatic AP shortening^36^. On the other hand, it is accepted that shortening of the APD leads to abbreviation of the refractory period, which fosters reentrant arrhythmias such as atrial fibrillation (AF)^37^. Therefore, we surmise that subjects carrying the p.P888L SAP97 polymorphism are more prone to develop AF, in the presence of either another mutation underlying familial AF or when co-existing with additional AF risk factors. Furthermore, dysregulation of I_to,f_ has also been implicated in early repolarization syndrome and mutations that increase in I_to,f_ are associated with Brugada Syndrome, an inherited cardiac arrhythmia syndrome^38^. Thus, it seems reasonable to speculate that the p.P888L polymorphism contributes to increase the expressivity of these syndromes. Finally, mathematical models and experiments in ventricular cardiomyocytes demonstrated that a large and slowly inactivating I_to_ can paradoxically exacerbate occurrence of early afterdepolarizations (EADs) and EADs-related arrhythmias in large mammals including humans^39–41^. Indeed, by lowering the plateau voltage during the early phase 1 of the AP plateau, I_to_ delays the subsequent activation of other slower time and voltage-dependent outward currents (mainly I_Ks_), thus diminishing their contribution to repolarization reserve during phases 2 and 3 of the AP, and thereby facilitating EADs. Therefore, the augmentation of ventricular I_to_ produced by p.P888L SAP97 might also be proarrhythmic by promoting EADs provided the repolarization reserve is compromised.

As mentioned, *DLG1* might act as a “modifier gene”^7^, i.e., a genetic factor capable of modifying the consequences of other disease-causing mutations. The p.P888L polymorphism is a common variant in the European population; however, the contribution of common variants to the genetic architecture of Mendelian inherited arrhythmias has been described and stressed^15^. Genetic factors also influence the burden of acquired arrhythmias like AF. Therefore, it would be of interest to analyze whether the electrophysiological effects of p.P888L SAP97 at the cellular level, imply electrocardiographic differences in subjects carrying the polymorphism and, if so, their possible repercussion in terms of arrhythmic risk.

We have combined experiments in an heterologous expression system and in a mouse model and each approach has its own advantages and limitations. AAV–mediated gene transfer in mice is a powerful technique that has wide potential for study of the consequences of diverse mutations in a cardiac specific manner. It allowed us to trans-express human SAP97 proteins without changing excessively the total level of production of intrinsic wildtype protein^16^, and to demonstrate in an *in vivo* model that the common p.P888L SAP97 polymorphism increases the I_to,f_ and abbreviates the APD and the QT interval. The approach empowered us to complete experiments in the whole animal and in isolated cardiomyocytes, while critically reducing the number of mice needed for the study, avoiding the crossbreeding steps necessary for the generation of knock-in mice^17^. However, one limitation is that the approach does not allow the analysis of the effects of a heterozygous variant. In addition, as discussed extensively above, neither the mouse model data nor the heterologous experiment results represent the human situation, and any extrapolation must be made under extreme caution.

Despite the above limitations, we feel confident to conclude that in mice the p.P888L SAP97 polymorphism markedly slows I_to,f_ inactivation kinetics increasing the I_to,f_ charge density via a CaMKII-dependent phosphorylation of Kv4.3 channels. These changes shorten APD and the QT interval and may increase the risk of arrhythmias.

## Methods

### Mouse model

Animal studies were approved by the Committees on the Use and Care of animals at the CNIC and the Complutense University and conform to the Guidelines from Directive 2010/63/EU of the European Parliament on the protection of animals used for scientific purposes. We generated cardiac-specific mice trans-expressing WT, p.P888L SAP97 or empty vector (Sham) using AAV-mediated gene delivery (Supplementary Fig. I)^16^. Ten to twenty weeks after infection with AAV particles, the expression of the transgene was stable, and animals were used for subsequent analyses.

#### Mouse ventricular myocyte isolation

Single mouse cardiomyocytes were isolated by enzymatic dissociation with collagenase type II (Worthington Biochemical Corporation Lakewood, NJ, USA) and protease (type XIV, Sigma Chemical Co. London, UK) from the three groups of mice following previously described methods^26^.

#### ECG recordings

We recorded four-lead surface ECGs in anaesthetized mice by using subcutaneous needle electrodes and an MP36R amplifier (BIOPAC Systems)^16^. An experienced investigator blinded to the study measured ECG parameters, including P wave, PR, QRS, QT, and RR interval using the Acknowledge 4.1 analysis software (BIOPAC Systems).

### Cell culture and transfection

Dr. Hugues Abriel (University of Bern, Switzerland) and Dr. Stéphane Hatem (Sorbonne University, France) kindly provided the cDNA encoding the human cardiac SAP97 isoform that contains the I3 but not the I1A domain (I3-I1A) tagged with ds-Red. We introduced the p.P888L variant using the QuikChange Site-Directed Mutagenesis kit (Stratagene, USA) as previously described^6,26^.

Chinese hamster ovary (CHO) cells were cultured as previously described^6,26,27^ and co-transfected with the cDNA encoding Kv4.3 channels and WT or p.P888L SAP97 using FUGENE XtremeGENE (Roche Diagnostics, Switzerland) according to manufacturer´s instructions.

Mouse fibroblasts or *Ltk*^−^ cells stably expressing hKv1.5 channels were cultured and transiently transfected with the cDNA encoding WT or p.P888L SAP97 using Lipofectamine 2000 (Invitrogen, USA) according to manufacturer´s instructions.

### Patch-clamping

Currents were recorded at 21–23 °C using the whole-cell patch-clamp technique and filtered at half the sampling frequency^6,21,26,27^. Series resistance was compensated manually and ≥80% compensation was achieved. Under our experimental conditions no significant voltage errors (<5 mV) due to series resistance were expected with the micropipettes used (with resistance <1.5 MΩ for I_Na_ and <3.5 MΩ for other currents). Action potentials (APs) were recorded at room temperature using the current-clamp configuration at a frequency of 1 Hz^26^. APs were elicited by 2-ms depolarizing current pulses and resistance of the micropipettes used was >7 MΩ.

### Western-blot (WB) analysis

SAP97, Nav1.5, and Kir2.1 proteins were detected in ventricular samples from Sham, WT or p.P888L trans-expressing mice by WB following previously described procedures^6,21,26,27^. Expression of Kv4.3, CaMKIIδ and phosphorylated-CaMKII (CaMKII-P) proteins was determined in CHO cells cotransfected with Kv4.3 and WT or p.P888L SAP97. In a subset of experiments, CaMKII was silenced in CHO cells by transfection of four different siRNA duplexes against the main four CaMKII isoforms expressed in CHO cells by using Lipofectamine 2000, according to manufacturer instructions. CaMKII silencing was confirmed by WB^6,26^.

### Blind protein-protein docking

A protein-protein docking was performed by means of the ClusPro web server (https://cluspro.org) to identify putative differences in the binding of CaMKIIδ to the SH3-GUK region induced by the presence of the Pro-to-Leu substitution at position 888 of SAP97.

### Statistical analysis

Results are expressed as mean ± SEM. Unpaired t-test or one-way ANOVA followed by Tukey’s test were used where appropriate. In small-size samples (n < 15), statistical significance was confirmed by using nonparametric tests. Data were also analyzed with multilevel mixed-effects models to take into account repeated sample assessments. A value of P < 0.05 was considered significant. Additional details are presented in Supplementary Methods.

## Supplementary information

Supplementary Information.

## Data Availability

The datasets analyzed during the current study are available from the corresponding author on reasonable request.
