# Supplementary material for: The p.P888L SAP97 polymorphism increases the transient outward current (Ito,f) and abbreviates the action potential duration and the QT interval
Source: Sci Rep. 2020 Jul 1;10:10707. doi: 10.1038/s41598-020-67109-z (PMC7329876; doi:10.1038/s41598-020-67109-z)
Supplement: Supplementary file 1 — Supplementary Information. [file 41598_2020_67109_MOESM1_ESM.pdf]

The p.P888L SAP97 polymorphism increases the transient outward current ($I_{to,f}$) and abbreviates the action potential duration and the QT interval

David Tinaquero, Teresa Crespo-García, Raquel G. Utrilla, Paloma Nieto-Marín, Andrés González-Guerra, Marcos Matamoros, Marta Pérez-Hernández, María Tamargo, Marcos Rubio-Alarcón, Anabel Cámara-Checa, María Dago, Jorge Cebrián, José Jalife, Juan Tamargo, Juan Antonio Bernal, Ricardo Caballero, Eva Delpón and the ITACA investigators.

Supplementary Material and Methods

1. Plasmids used and Site-Directed mutagenesis. Human SAP97 cDNA subcloned in pIRES2-dsRed1 encoding the cardiac SAP97 isoform that contains the I3 but not the I1A domain (I3-I1A) was kindly provided by Dr. Hugues Abriel (University of Bern, Switzerland) and Dr. Stéphane Hatem (Sorbonne University, France) [1]. The p.P888L variation was introduced by using the QuikChange Site-Directed Mutagenesis kit (Stratagene, USA) as previously described [2,3]. On the other hand, to eliminate the primary site for CaMKII phosphorylation the p.S550A substitution was introduced in the rat Kv4.3 cDNA subcloned in pBKCMV as previously described [4]. In all cases, the substitutions were confirmed by Sanger sequencing.

2. Mouse model.

The experiments with animals conformed to the guidelines from Directive 2010/63/EU of the European Parliament on the protection of animals used for scientific purposes. All animals were maintained and handled according to CNIC Institutional Ethics Committee recommendations (PROEX 019/17) and the Complutense University Committee on the Use and Care of Animals (SAF2017-88116P). Wild-type 4-6-week-old male C57BL/6J mice were obtained from Charles River Laboratories. Mice were individually housed in wire-bottomed cages in a temperature-controlled room (22±0.8°C) with a 12h light–dark cycle and a relative humidity of 55±10%. The mice had free access to food and water.

Three cardiac–specific transgenic-like mouse models on the basis of adeno-associated virus (AAV) gene transfer [5] were created trans-expressing or not, wild type (WT) and p.P888L SAP97, respectively. To this end AAV vectors driven from the cardiomyocyte-specific cardiac troponin T proximal promoter and encoding or not (empty vector [Sham]), WT human I3-I1A SAP97 (SAP97 WT) or p.P888L SAP97 were constructed. The use of the cardiac troponin T proximal promoter guarantees the expression of the delivered genes mostly in the heart [5].

AAV vector production and purification. AAV vectors were all produced by the triple transfection method, using HEK 293T cells as described previously [5]. AAV plasmids were cloned and propagated in the Stbl3 *E. coli* strain (Life Technologies). Shuttle plasmids pAAV-control (empty vector), pAAV-SAP97 WT and pAAV-SAP97 p.P888L (Supplementary Fig. I) were derived from pAcTnT (a gift from Dr B.A. French; University of Virginia, USA) and packaged into AAV-9 capsids with the use of helper plasmids pAdDF6 (providing the three adenoviral helper genes) and pAAV2/9 (providing rep and cap viral genes), obtained from PennVector. The AAV shuttle and helper plasmids were transfected into HEK 293T cells by calcium-phosphate co-precipitation. A total of 840 µg of plasmid DNA (mixed in an equimolar ratio) was used per Hyperflask (Corning) seeded with $1.2x10^8$ cells the day before. Seventy-two hours after transfection, the cells were collected by centrifugation and the cell pellet was resuspended in TMS (50 mM Tris HCl, 150 mM NaCl, 2 mM $MgCl_2$) on ice before digestion with DNase I and RNaseA (0.1 mg/mL each; Roche) at 37 °C for 60 minutes. Clarified supernatant containing the viral particles was obtained by iodixanol gradient centrifugation. Gradient fractions containing virus were concentrated using Amicon UltraCel columns (Millipore) and stored at -70ºC.

Determination of AAV vector titer. Titers for the AAV vectors (vg per mL) were determined by quantitative real-time PCR as described [5]. Known copy numbers (10^5–10^8) of the respective plasmid (pAAV empty vector, pAAV-SAP97 WT and pAAV-SAP97 p.P888L) carrying the appropriate cDNA were used to construct standard curves.

Four- to 6-week-old wild-type C57BL6/J male mice were injected with 3.5 × 10^{10} viral genomes encoding the empty vector, WT or p.P888L SAP97 through the intravenous femoral route. This method ensures the expression of the protein in the cardiac tissue for more than 6 months, a period of time in which mice did not show any apparent illnesses or increased mortality. Importantly, the use of this mouse model avoids the maintenance of large colonies of genetically modified animals and, thus, decreases the number of animals used, which fits with public concerns and the minimal-use concept expressed in the 3 Rs (3Rs) principle for the rational use of animals in research: Replacement of animals by alternatives wherever possible; Reduction in the number of animals used; and Refinement of experimental conditions and procedures to minimize the harm to animals. Ten to twenty weeks after infection with AAV particles, animals were used for the subsequent analyses (ECG or cellular electrophysiological recordings, and protein expression).

Surface ECG. Mice were anaesthetized using isoflurane inhalation (0.8-1.0% volume in oxygen), and efficacy of the anaesthesia was monitored by watching breathing speed. Four-lead surface ECGs were recorded, for a period of 5 minutes, from subcutaneous 23-gauge needle electrodes attached to each limb using the MP36R amplificator unit (BIOPAC Systems) [5]. ECG parameters such as the duration of the P wave, and of the PR, QRS, QT, and RR intervals were measured by using the Acknowledge 4.1 analysis (BIOPAC Systems) software.

Mouse ventricular myocyte isolation. Single ventricular myocytes were isolated by enzymatic dissociation with collagenase type II (Worthington Biochemical Corporation Lakewood, NJ, USA) and protease (type XIV, Sigma Chemical Co. London, UK) as described [6,7,8]. Mice were heparinized (5.000 U/kg i.p.) and anesthetized with ketamine (150 mg/kg i.p.) and xylazine (10 mg/kg i.p.).

3. **Cell culture and transfection.** Chinese Hamster Ovary (CHO) cells (ATCC, LGC Standards, UK) were cultured in 60 mm dishes at 37ºC in an atmosphere of 5% of CO_2, with humidity of ≈95%, as previously described [2,3,6-9]. CHO cells were transfected with the cDNA encoding Kv4.3 (3 μg) and

cotransfected or not with the cDNA encoding WT, or p.P888L SAP97 (1.6 μg) plus the cDNA encoding the CD8 antigen (0.5 μg) using FUGENE XtremeGENE (Roche Diagnostics, Switzerland) according to the manufacturer´s instructions [2,3,6-9]. Forty eight h after transfection, cells were incubated with polystyrene microbeads precoated with anti-CD8 antibody (Dynabeads M450; Life Technologies). Most of the cells that were beaded also had channel expression. Mouse fibroblasts or *Ltk⁻* cells stably expressing hKv1.5 channels were grown in Dulbecco's modified Eagle medium (Invitrogen, USA) supplemented with 10% horse serum, 0.05 mg/mL gentamicin, and 0.25 mg/mL G418 (a neomycin analog; Gibco, USA) in a 5% CO_2 atmosphere [7,10]. *Ltk⁻* cells were transfected or not [SAP97 (-)] with the cDNA encoding WT or p.P888L SAP97 (1.6 μg) using Lipofectamine 2000 (Thermofisher Scientific, USA) according to the manufacturer´s instructions. In all cases the expression of WT or p.P888L SAP97 was identified by the red fluorescent signal under fluorescent microscopy (Nikon Eclipse TE2000S, Nikon). To minimize the influence of the expression variability of transiently transfected mammalian cell lines, each construct was tested in a large number of cells obtained from at least three different transfection batches.

4. Patch-clamping. Currents were recorded at room temperature (21-23ºC) by means of the whole-cell patch-clamp technique using an Axopatch-200B patch clamp amplifier (Molecular Devices, USA [2,3,6-16]. Recording pipettes were pulled from 1.0 mm o.d. borosilicate capillary tubes (GD1, Narishige Co., Ltd, Japan) using a programmable patch micropipette puller (Model P-2000 Brown-Flaming, Sutter Instruments Co., USA) and were heat-polished with a microforge (Model MF-83, Narishige). Micropipette resistance was kept below 1.5 MΩ for I_{Na}, below 3.5 MΩ for the rest of the currents or above 7 MΩ for action potentials when filled with the internal solution and immersed in the external solution. In all the experiments, series resistance was compensated manually by using the series resistance compensation unit of the Axopatch amplifier, and ≥80% compensation was achieved.

The remaining access resistance after compensation and cell capacitance were 1.3±0.8 MΩ and 10.7±0.5 pF (n=80) and 1.4±0.7 MΩ and 10.4±0.8 pF (n=40) in *Ltk*- and CHO cells, and 2.5±1.3 MΩ and 125±2.9 pF (n=222) in ventricular myocytes from mice, respectively. Therefore, under our experimental conditions no significant voltage errors (<5 mV) due to series resistance were expected with the micropipettes used. To minimize the contribution of time-dependent shifts of channel availability during I_{Na} recordings, all data were collected 5-10 min after establishing the whole-cell configuration. Under these conditions current amplitudes and voltage dependence of activation and inactivation were stable during the time of recordings [2,3,8,9,12,15]. The current recordings were sampled at 4 kHz (except for I_{Na} that was sampled at 50 kHz), filtered at half the sampling frequency and stored on the hard disk of a computer for subsequent analysis.

Solutions (mouse ventricular myocytes). To record I_{Na} the external solution contained (mM): NaCl 4, $MgCl_2$ 1, $CaCl_2$ 1, $CdCl_2$ 0.1, CsCl 133.5, HEPES 20, glucose 11 (pH=7.35 with CsOH). Recording pipettes were filled with an internal solution containing (mM): NaF 10, CsF 110, CsCl 20, HEPES 10, and EGTA 10 (pH 7.35 with CsOH). To record I_{K1}, the external solution contained (mM): NaCl 140, KCl 4, $CaCl_2$ 1, $MgCl_2$ 1, HEPES 10, 4-aminopyridine 2, glucose 10, nifedipine (1 μM), atropine (0.1 μM), and glibenclamide (10 μM) (pH=7.4 with NaOH). Recording pipettes were filled with an internal solution containing (mM): K-aspartate 80, KCl 42, KH_2PO_4 10, MgATP 5, phosphocreatine 3, HEPES 5, and EGTA 5 (pH 7.2 with KOH). I_{K1} current-voltage curves were corrected according to the calculated liquid junction potential (LJP=-13.2 mV) between the pipette and external solution [2,3,8]. To record outward K^+ currents the external and internal solutions were identical to those for I_{K1} recordings, but without 4-aminopyridine in the external solution. In some experiments, the Ca^{2+}-independent transient outward current ($I_{to,f}$) was recorded as the current resistant to 25 mM tetraethylammonium (TEA) [17]. This solution was prepared by equimolar substitution of NaCl by TEA in the external solution mentioned above. To record action potentials the external solution contained (mM): NaCl 136, KCl 4, $CaCl_2$ 1.8, $MgCl_2$ 1, HEPES 10, and glucose 10 (pH 7.4 with NaOH). The internal solution contained (mM): K-

aspartate 80, KCl 42, KH_2PO_4 10, MgATP 5, phosphocreatine 3, HEPES 5, and EGTA 5 (pH=7.2 with KOH).

Solutions (CHO and Ltk⁻ cells). To record Kv4.3 ($I_{Kv4.3}$) and Kv1.5 ($I_{Kv1.5}$) currents, cells were perfused with an external solution containing (mM): NaCl 136, KCl 4, $CaCl_2$ 1.8, $MgCl_2$ 1, HEPES 10, and glucose 10 (pH 7.4 with NaOH). In both cases, recording pipettes were filled with an internal solution containing (mM): K-aspartate 80, KCl 42, KH_2PO_4 10, MgATP 5, phosphocreatine 3, HEPES 5, and EGTA 5 (pH 7.2 with KOH). In some experiments, the effects of the CaMKII inhibitor autocamtide-2-related inhibitory peptide (AIP) were analyzed. This compound is not diffusible across cell membranes, and, thus, it was added to the internal solution that dialyzes the cells [9]. In these experiments, the tip of the pipette was filled with AIP-free internal solution, in order to obtain "control" current records.

Pulse protocols (mouse ventricular myocytes). To construct the current-voltage relationships for I_{Na}, 50-ms pulses in 5 mV increments from -120 mV to potentials between -100 and +30 mV were applied. To construct the inactivation curves, I_{Na} was recorded by applying 500-ms pulses from -120 mV to potentials between -140 and -20 mV in 10 mV increments followed by a test pulse to −40 mV. To analyze the recovery from inactivation of I_{Na}, two 50-ms pulses (P1 and P2) from –120 to -40 mV were applied at increasing coupling intervals (0.05-500 ms). A monoexponential function was fitted to the data to measure the reactivation kinetics.

The protocol to record I_{K1} consisted of 250-ms steps that were imposed in 10 mV increments from -80 mV to potentials ranging -120 and -40 mV. I_{K1} was always measured at the end of the 250-ms pulses. Current-voltage relationships for $I_{to,f}$ were constructed by applying 500-ms pulses in 10 mV increments from -80 mV to potentials ranging -120 and +50 mV. The $I_{to,f}$ was measured as the difference between peak current and the current at the end of the 500-ms pulse and the $I_{to,f}$ charge was estimated from the integral of the current traces comprising the area between the peak and the current at the end of the 500-ms pulse [13]. In another group of experiments, the $I_{to,f}$ and the rapidly activating slowly inactivating component ($I_{K,slow}$) were recorded by applying 4-s pulses from a holding potential of -80 mV to potentials

ranging -90 and +50 mV in 10 mV increments. A biexponential function was fitted to the decay of currents generated by pulses to +50 mV. As previously described [17], under these conditions the amplitude of the fast component of the exponential can be identified as the amplitude of the $I_{to,f}$, whereas the amplitude of the slow component can be identified as that of the $I_{K,slow}$. Furthermore, the fast and slow time constants of current decay were assimilated as the inactivation time constants of the $I_{to,f}$ and the $I_{K,slow}$, respectively. Superfusion of TEA (25 mM) inhibited $I_{K,slow}$ and I_{ss} currents by ~60%, leaving the $I_{to,f}$ unaffected [17].

Inactivation curves for $I_{to,f}$ were constructed by applying 500-ms pulses in 10 mV increments from -80 mV to potentials ranging -120 and +50 mV followed by a test pulse to +50 mV. To analyze the recovery from inactivation of $I_{to,f}$, two 500-ms pulses (P1 and P2) from –80 to +50 mV were applied at increasing coupling intervals (5-4000 ms). A monoexponential function was fitted to the data to measure $I_{to,f}$, reactivation kinetics in myocytes isolated from Sham and WT SAP97 mice. $I_{to,f}$, reactivation data from p.P888L SAP97 mice were fitted by a biexponential function.

Pulse protocols (CHO cells). To obtain current-voltage relationships for $I_{Kv4.3}$, 500-ms pulses in 10 mV increments from -80 mV to potentials between -90 and +50 mV were applied. $I_{Kv4.3}$ was measured as the maximum peak amplitude at each potential. $I_{Kv4.3}$ was also measured as the total charge crossing the membrane estimated from the integral of the current traces elicited at each potential. To obtain the inactivation curves for $I_{Kv4.3}$, a two-step protocol was used consisting of a first 500-ms conditioning pulse from –80 mV to potentials between –90 and +50 mV, followed by a test pulse to +50 mV. To analyze the recovery from inactivation of Kv4.3 channels, two 500-ms pulses (P1 and P2) from –80 to +50 mV were applied at increasing coupling intervals (5-4000 ms). A monoexponential function was fitted to the data (P2/P1 plotted as a function of the time interval) to obtain the time constant that defines the process.

Activation/conductance voltage curves for $I_{Kv4.3}$ recorded in CHO cells and for I_{Na} and $I_{to,f}$ recorded in mouse ventricular myocytes were constructed by plotting the normalized conductance as a function of the membrane potential. The conductance was estimated for each experiment by the equation:

$$G=I/(V_m-E_{rev})$$

where G is the conductance at the test potential V_m, I represents the peak maximum current at V_m, and E_{rev} is the reversal potential. To determine the E_{rev}, I_{Na} density-voltage relationships obtained in each experiment were fitted to a function of the form:

$$I=(V_m-E_{rev})*G_{max}*(1+exp[V_m-V_h]/k)^{-1}$$

where I is the peak current elicited at the test potential V_m, G_{max} is the maximum conductance, and k is the slope factor.

For $I_{Kv4.3}$ and $I_{to,f}$, the E_{rev} introduced for the calculation of the conductance was the value described in previous studies [14].

This procedure yields conductance curves for $I_{Kv4.3}$ and $I_{to,f}$ with less steep slopes than those obtained when the voltage dependence of the activation is measured with the full envelopes of tails. However, it is a procedure widely used for the comparative analysis of the effects on the voltage-dependence of activation [1,14].

Activation/conductance-voltage and inactivation curves were fitted with a Boltzmann distribution according to the following equation:

$$y = A /\{1 + exp [(V_h-V_m)/k]\})$$

where A is the amplitude term, V_h is the midpoint of activation/inactivation (in mV), V_m is the test potential and k represents the slope factor for the curve.

In each experiment, current amplitudes were normalized to membrane capacitance to obtain current densities. Action potentials were recorded using the current clamp configuration and elicited by depolarizing-current pulses of 2 ms in duration at 1.5 times the current threshold at a frequency of 1 Hz.

Pulse protocols (Ltk⁻ cells). The protocol used to obtain $I_{Kv1.5}$ current–voltage relationships consisted of 500-ms pulses in 10 mV increments from a holding potential of -80 mV to potentials ranging -80 and +60 mV. Between −80 and −40 mV, only passive linear leak was observed and least-squares fits to these data were used for passive leak correction. Deactivating 'tail' currents were recorded on return to

−40 mV. The activation curves were constructed by plotting tail current amplitude as a function of the membrane potential and were fitted with a Boltzmann function (see above). A monoexponential function was fitted to the current traces at +50 mV to obtain the time constant of activation and inactivation and to the tail current at -40 mV after pulses to +50 mV to determine the time course of deactivation.

5. Western-blot analysis.

Detection of SAP97, Nav1.5, and Kir2.1 proteins was carried out in ventricular samples from Sham, WT or p.P888L SAP97 mice by Western-blot following previously described procedures [2,3,6,8,9,11,16]. After excision, ventricles were washed in PBS, immediately snap-frozen in liquid nitrogen and stored at −80ºC. Ventricular tissue was homogenized in RIPA buffer (in mM): Tris-HCl 50, NaCl 150, and 1% NP-40, 0.5% sodium-deoxicolate and protease inhibitor cocktail (Sigma) using a loose-fitting Dounce homogenizer (10 strokes) and an UltraTurrax T-25 tissue grinder (3x10 s bursts). Nuclei and cell debris were removed by centrifugation at 12.000 rpm for 20 min at 4ºC. Expression of Kv4.3, CaMKIIδ and phosphorylated-CaMKII (CaMKII-P) proteins was determined in CHO cells cotransfected with Kv4.3 and WT or p.P888L SAP97 as described above. Transfected CHO cells were collected in RIPA buffer. Nuclei and cell debris were removed by centrifugation at 14.000 rpm for 20 min at 4ºC. In both cases (ventricular tissue and CHO cells) the total protein amount of the extracts was calculated with the bicinchoninic acid method (BCA, Pierce, USA). Afterwards, samples were run on 7.5% (Nav1.5) or 10% (Kir2.1, SAP97, Kv4.3, CaMKII, and CaMKII-P) Mini-PROTEAN TGX™ stain-free gels (Bio-Rad, USA) and transferred to nitrocellulose membranes. Nonspecific binding sites were blocked with 5% nonfat dried milk in PBS with Tween-20 (0.05%) for 1 hour at room temperature. Membranes were then incubated with rabbit polyclonal anti-Nav1.5 (1:1000; ab56240 Abcam, UK), rabbit monoclonal anti-Kir2.1 (1:1000, ab109750 Abcam), rabbit monoclonal anti-SAP97 (1:1000, ab134156 Abcam), mouse monoclonal anti-Kv4.3 (1:500; clone K75/41 Neuromab, USA), rabbit polyclonal anti-CaMKIIδ (1:5000; A010-56AP Badrilla, UK) or rabbit polyclonal anti-CaMKII-P (1:750; ab32678 Abcam) antibodies

overnight at 4ºC. These antibodies bind to residues 1857-1906 (intracellular C-terminal domain) of human Nav1.5, residues 107-122 (extracellular domain) of human Kir2.1 channels, residues located at the N-terminal domain of SAP97, residues 415-636 (cytoplasmic C-terminus) of human Kv4.3, residues 485-499 (C-terminus) of human CaMKIIδ, and the phosphorylated Thr286 residue of CaMKII-P, respectively. Afterwards, samples were incubated for 1 hour with peroxidase-conjugated goat anti-rabbit secondary antibody (1:10,000; Jackson Immunoresearch, USA). In some experiments, fluorescent-dye conjugated secondary antibodies (anti-rabbit Starbright 700 1/2500, Bio-Rad) were used. Membranes were washed three times with PBS-Tween after adding primary and secondary antibodies. Protein expression was detected by chemiluminescence (ECL, General Electric Healthcare, USA) and visualized using the Chemidoc MP System and Image Lab 5.2.1. software (Bio-Rad). When the fluorescent-dye conjugated secondary antibodies were used, protein expression was visualized by using the LED imaging modules of the Chemidoc MP System. As loading control the total protein was used by means of stain-free gels (Bio-Rad) and ImageLab software [2,3]. Expression of the proteins in the immunoblot was then normalized to total protein. In some experiments Na^+-K^+ ATPase, detected by mouse monoclonal anti alpha1 Na^+-K^+ ATPase antibody (1:1000, ab7671 Abcam) was used as a loading control.

For CaMKII silencing, CHO cells (transfected with Kv4.3 and WT or p.P888L SAP97 24 h before) were co-transfected with four different siRNA duplexes (100 nM) or with siRNA Universal Negative Control (scrambled, Sigma) by using Lipofectamine 2000 (Invitrogen, USA), according to manufacturer instructions [3,8]. The sequence of the siRNA duplexes against the main four CaMKII isoforms expressed in CHO cells are the following: (CaMKIIα [XM_007646829.1] sense: 5'-GUUCCAGCGUUCAGUUAAU-3'; antisense: 5'-AUUAACUGAACGCUGGAAC-3'. CaMKIIβ [XM_007645266.2] sense: 5'-CUCAUUUGAGCCUGAAGCU-3'; antisense: 5'-AGCUUCAGGCUCAAAUGAG-3'. CaMKIIδ [XM_007653648.2] sense: 5'-CGUAAAGAUCCUUAUGGAA-3'; antisense: 5'-UUCCAUAAGGAUCUUUACG-3'. CaMKIIγ

[XM_007652669.2] sense: 5'-CUGUAACACCACUACAGAA-3'; antisense: 5'-UUCUGUAGUGGUGUUACAG-3'). Silencing was confirmed by Western-blot by using rabbit polyclonal anti-CaMKIIδ (1:5000; A010-56AP Badrilla). Electrophysiological and Western-blot analyses were performed 24 h after siRNA transfection.

6. Blind protein-protein docking. A protein-protein docking was performed by means of the ClusPro web server (https://cluspro.org) to identify putative differences in the binding of CaMKIIδ to the SH3-GUK region induced by the presence of the Pro-to-Leu substitution at position 888 of SAP97 [18]. This approach has been widely used for similar purposes by many investigators. ClusPro uses Fast Fourier Transform-based PIPER for rigid body docking. The server performs three computational steps: 1) rigid body docking by sampling billions of conformations, 2) root-mean-square deviation (RMSD) based clustering of the 1000 lowest energy structures generated to find the largest clusters that will represent the most likely models of the complex, and 3) refinement of selected structures using energy minimizations. For the docking, the crystallized structures of the SH3-GUK domain of SAP97 (PDB ID 3UAT) and of CaMKIIδ (PDB ID 6AYW) were used. The crystal structure of the SH3-GUK domain (spanning residues between 582 and 908) of SAP97 (Q62696) was reported by Zhu et al [19]. In this structure, the proline that is equivalent to Pro-888 is located at position 906. The Pro-to-Leu substitution at this position was performed by means of the mutagenesis wizard of PyMOL (The PyMOL Molecular Graphics System, Version 2.0 Schrödinger, LLC). This procedure allowed us to obtain a new PDB file corresponding to the mutated form of the SH3-GUK domain (named as p.P888L for consistency). The crystallized structure of CaMKIIδ (Q13557) was released by Somoza and Villaseñor in 2017 (10.2210/pdb6AYW/pdb) and includes a dimer of chains A and B (spanning residues 3 and 300). The SAP97 SH3-GUK domain (WT or p.P888L) was chosen as the receptor and placed at the origin of the coordinate system on a fixed grid, whereas CaMKIIδ was selected as the ligand and placed in a movable grid. When the model running is completed, the web server provides the PDB files and measured parameters of the 30 most populated clusters (Supplementary Table III). For each cluster,

Cluspro yields the size (number of conformations/members), the weighted energy score of the cluster center (i.e. the structure that has the highest number of neighbour structures in the cluster), and the energy score of the lowest energy structure in the cluster. The model is based on cluster size rather than on energy. Indeed, low energy regions tend to generate large clusters of docked structures, and the size of a cluster is approximately proportional to its probability, therefore, the higher the size, the more likely the conformation of the complex.

7. Statistical analysis. Results are expressed as mean±SEM. Unpaired t-test or one-way ANOVA followed by Tukey's test were used where appropriate. In small-size samples (n<15), statistical significance was confirmed by using nonparametric tests. To take into account repeated sample assessments, data were analyzed with multilevel mixed-effects models. Comparisons between categorical variables were done using Fisher´s exact test. A value of P<0.05 was considered significant.

SUPPLEMENTARY FIGURES

Supplementary Fig. I. Expression of SAP97 in three cardiac–specific transgenic-like mouse models on the basis of adeno-associated virus (AAV) gene transfer. (a) Schematic diagram of the AAV vectors encoding human WT (pAcTnT-SAP97WT-IRES-tdTomato) or the p.P888L (pAcTnT-p.P888L-IRES-tdTomato) SAP97 variant driven by the cardiac troponin T (cTnT) proximal promoter and followed by tandem dimeric Tomato protein (tdTomato) after an internal ribosome entry site (IRES). AAV inverted terminal repeats (ITR) and the poly-adenylation sequence (pA) are also indicated. (b) Representative fluorescence microscopy image of a AAV transduced heart showing the characteristic viral vector mosaic cellular distribution of tdTomato expression. To obtain the fluorescent images a Nikon A1-R inverted confocal microscope was used. The quantification of the fluorescence intensity of the transduced protein expression allowed to assign the number of integrated viral genomes per cardiomyocyte. The percentage of positive cells in the total tissue area was calculated after segmentation of the cells and measurement of signal intensities by means of ImageJ. The data obtained indicated that the transgene was homogeneously distributed throughout the heart without differences among chambers and with a cardiomyocyte transduction rate of ≈95% [5]. (c) Densitometric analysis of the expression of SAP97 protein detected by Western-blot in ventricular samples obtained from Sham, WT or p.P888L SAP97 transexpressing mice. Bars are the mean±SEM of samples from ≥3 mice per group. *P<0.05 vs Sham mice. Western-blot experiments showed that the expression of SAP97 protein increased by ≈50% in mice expressing AAV encoding WT and p.P888L SAP97 compared to Sham animals.

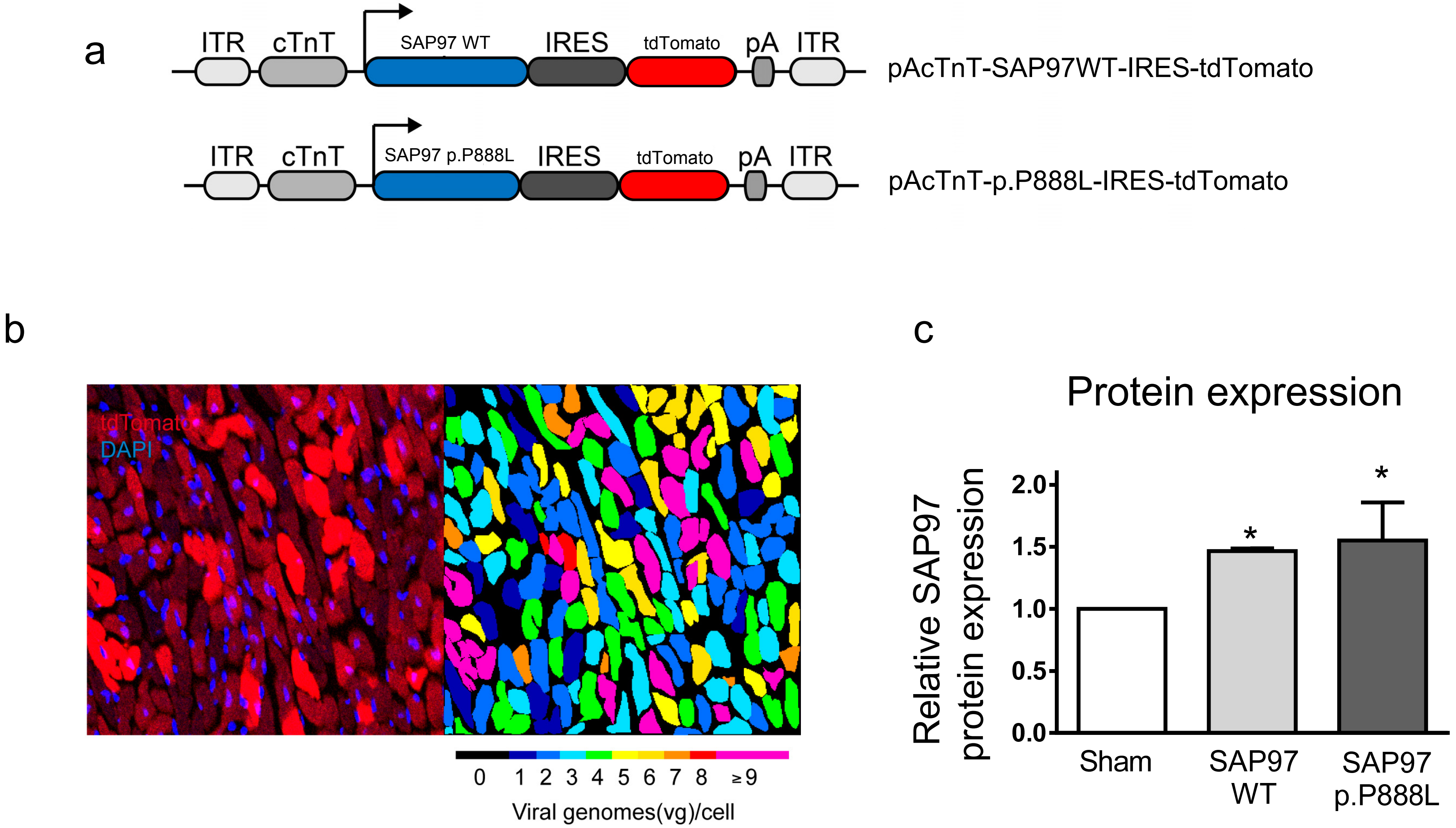

Supplementary Figure I

Supplementary Fig. II. WT and p.P888L SAP97 increase cardiac Kv1.5 currents. (a) Families of $I_{Kv1.5}$ traces recorded by applying 500-ms pulses from a holding potential of -80 mV to potentials between -80 and +60 mV in 10 mV steps followed by repolarization to -40 mV to record the tail currents in *Ltk*⁻ cells expressing hKv1.5 channels co-transfected or not [(-)] with WT or p.P888L SAP97. (b) Mean current density-voltage curves for $I_{Kv1.5}$ recorded in the 3 experimental groups. In panel b, each point represents the mean±SEM of "n" experiments/cells. *P<0.05 vs (-).

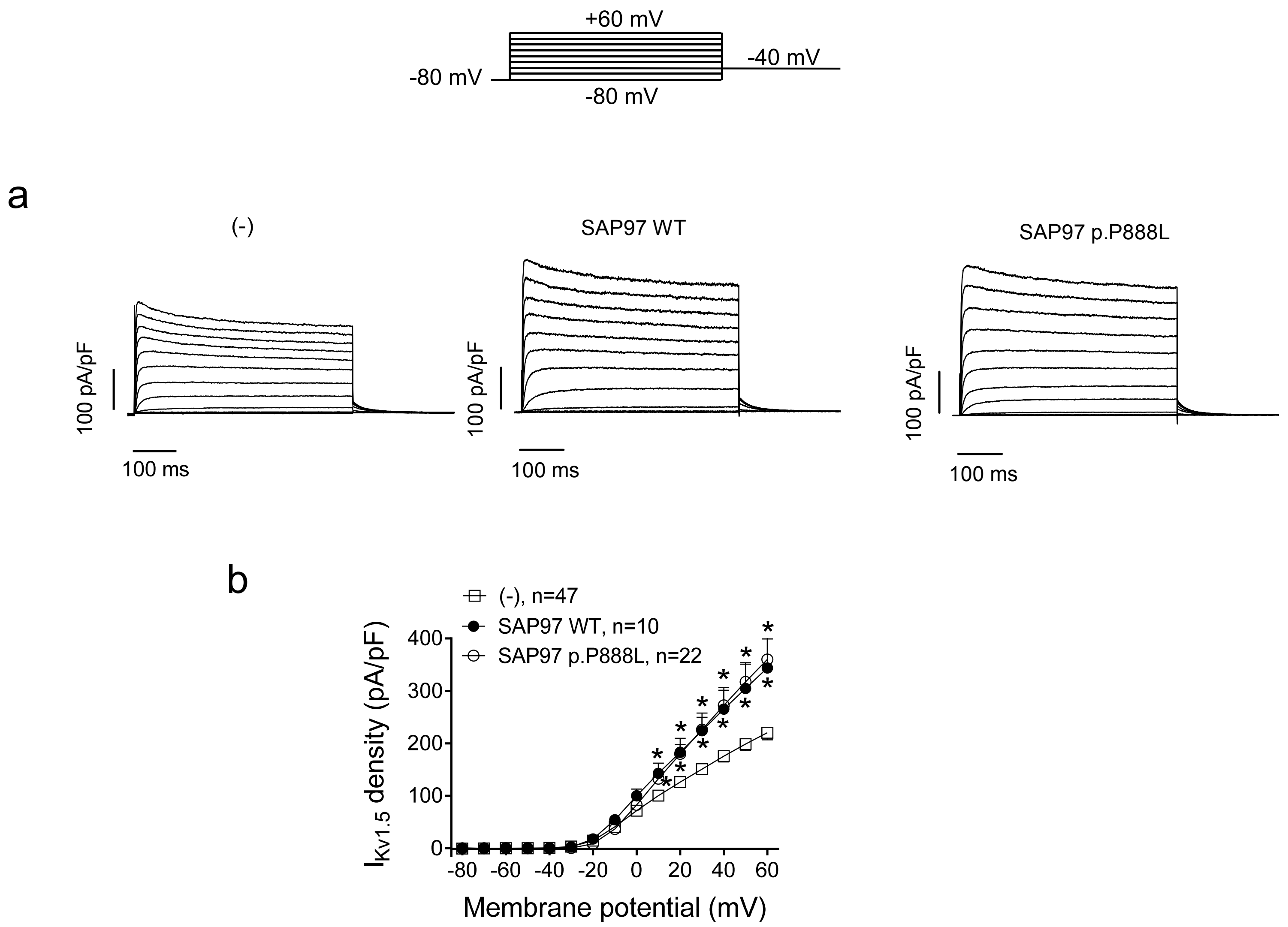

Supplementary Figure II

Supplementary Fig. III. CaMKII inhibition suppresses the effects of p.P888L SAP97 on Kv4.3 channels. (a) Western-blot image (left) and its corresponding stain-free gel (right) showing CaMKII expression in CaMKII-silenced CHO cells or in cells transfected with scrambled (Control) siRNA. (b) Mean densitometric analysis of CaMKII levels normalized to total protein. (c and e) Superimposed $I_{Kv4.3}$ traces recorded in two cells expressing Kv4.3 channels together with p.P888L (c) or WT (e) incubated or not with KN93 (1 μM) for 24 h. (d and f) $I_{Kv4.3}$ charge density-voltage relationship recorded in CHO cells expressing Kv4.3 channels co-expressed with p.P888L (d) or WT (f) SAP97 incubated or not with KN93. In d and f, *P<0.05 vs cells non-incubated with KN93. (g) Superimposed normalized $I_{Kv4.3}$ traces generated by Kv4.3 channels co-transfected with p.P888L SAP97 just after the patch rupture (first) and after the effects produced by the intracellular dialysis with AIP reached steady-state. (h) Percentage of AIP-induced reduction of the charge measured by the integration of $I_{Kv4.3}$ traces generated by pulses to +50 mV in CHO cells expressing Kv4.3 co-transfected with WT or p.P888L SAP97. *P<0.05 vs WT SAP97. In b and h, each bar represents the mean±SEM of the experiments shown as dots. In d and f, each point represents the mean±SEM of "n" experiments.

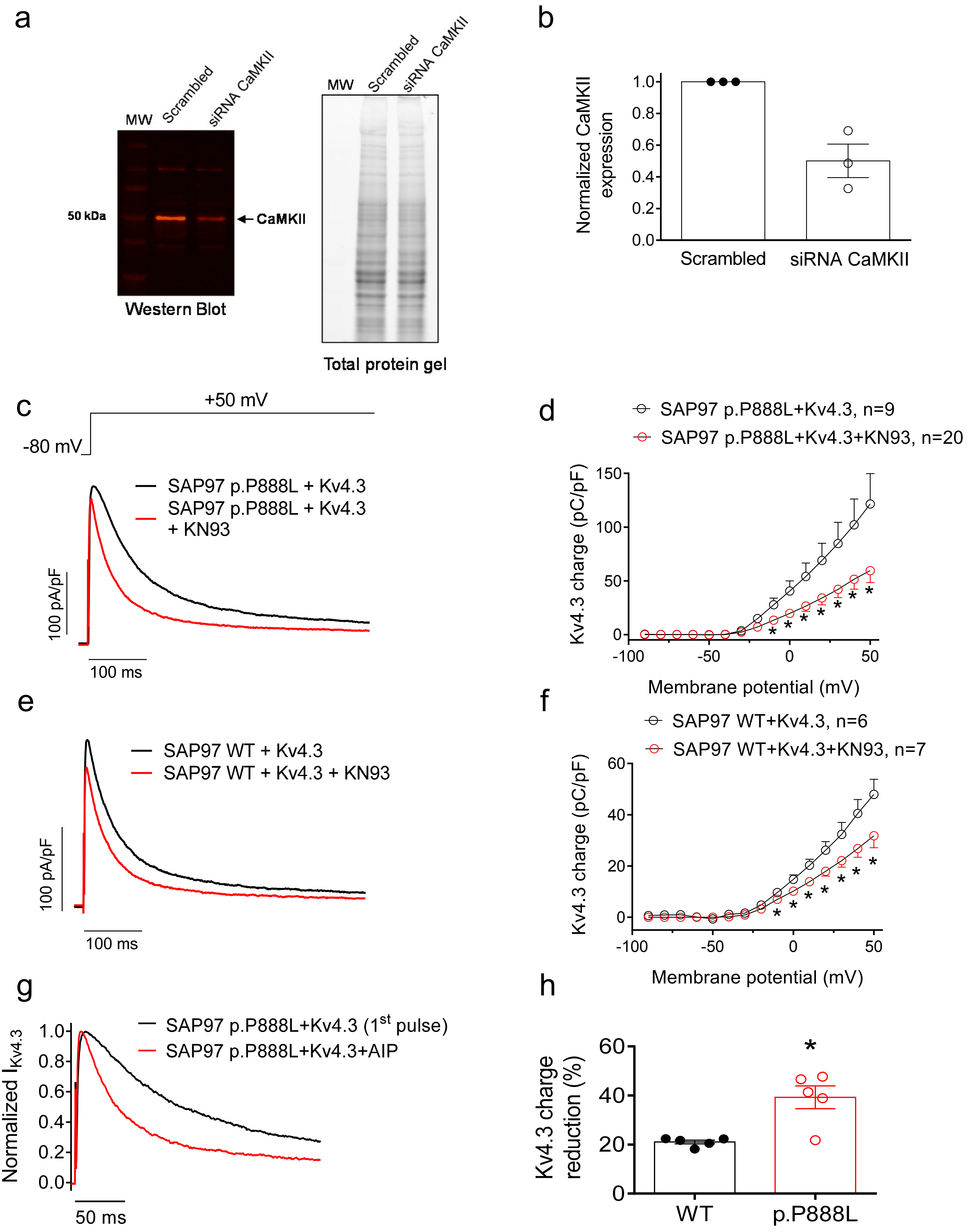

Supplementary Figure III

Supplementary Fig. IV. Full length uncropped membrane and total protein gel of WB image shown in Fig. 3e.

Supplementary Figure IV

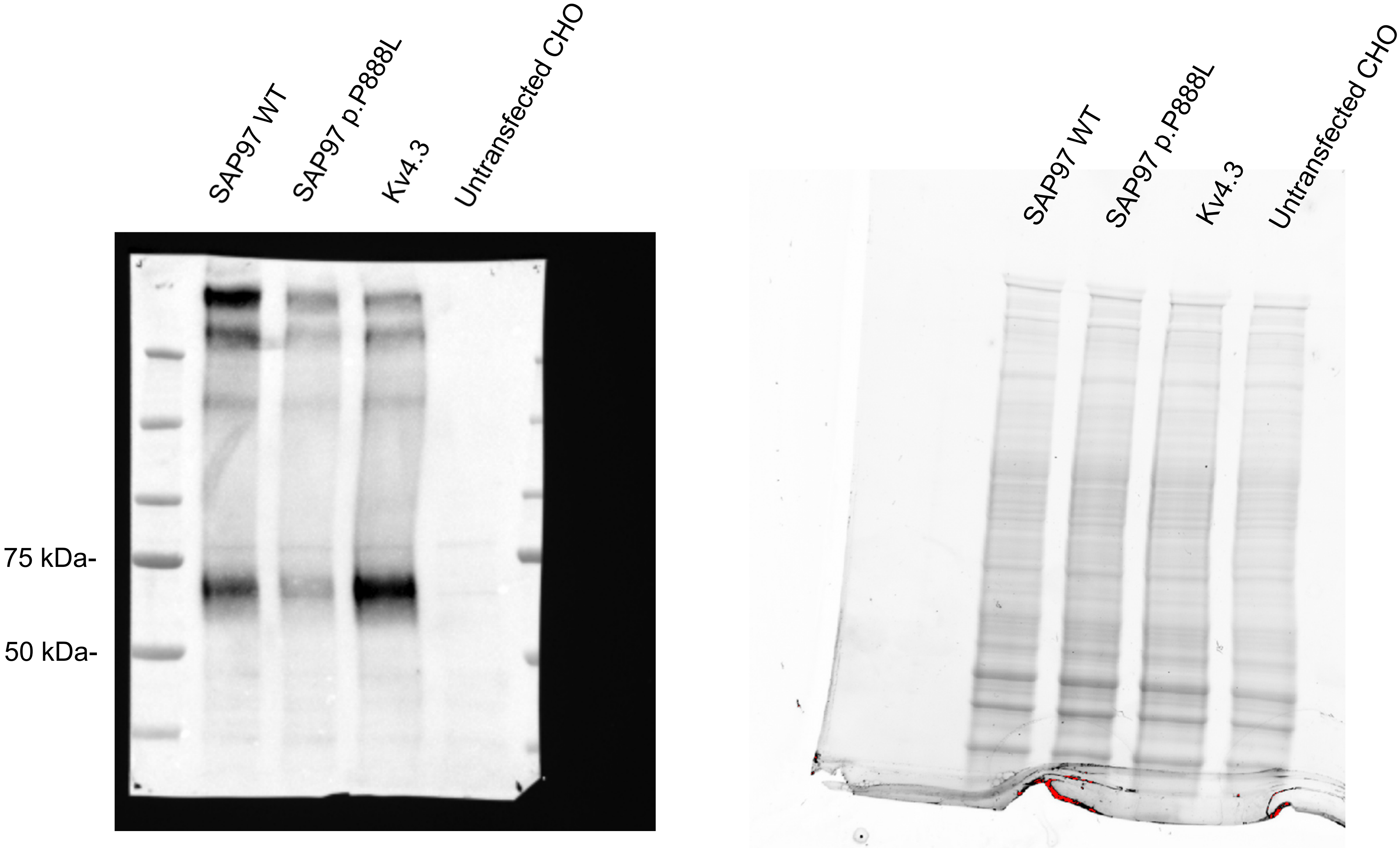

WB Kv4.3

Total protein gel

Supplementary Fig. V. (a and b) Full length uncropped membranes and total protein gels of WB images shown in Fig. 5a (panel a) and 5c (panel b).

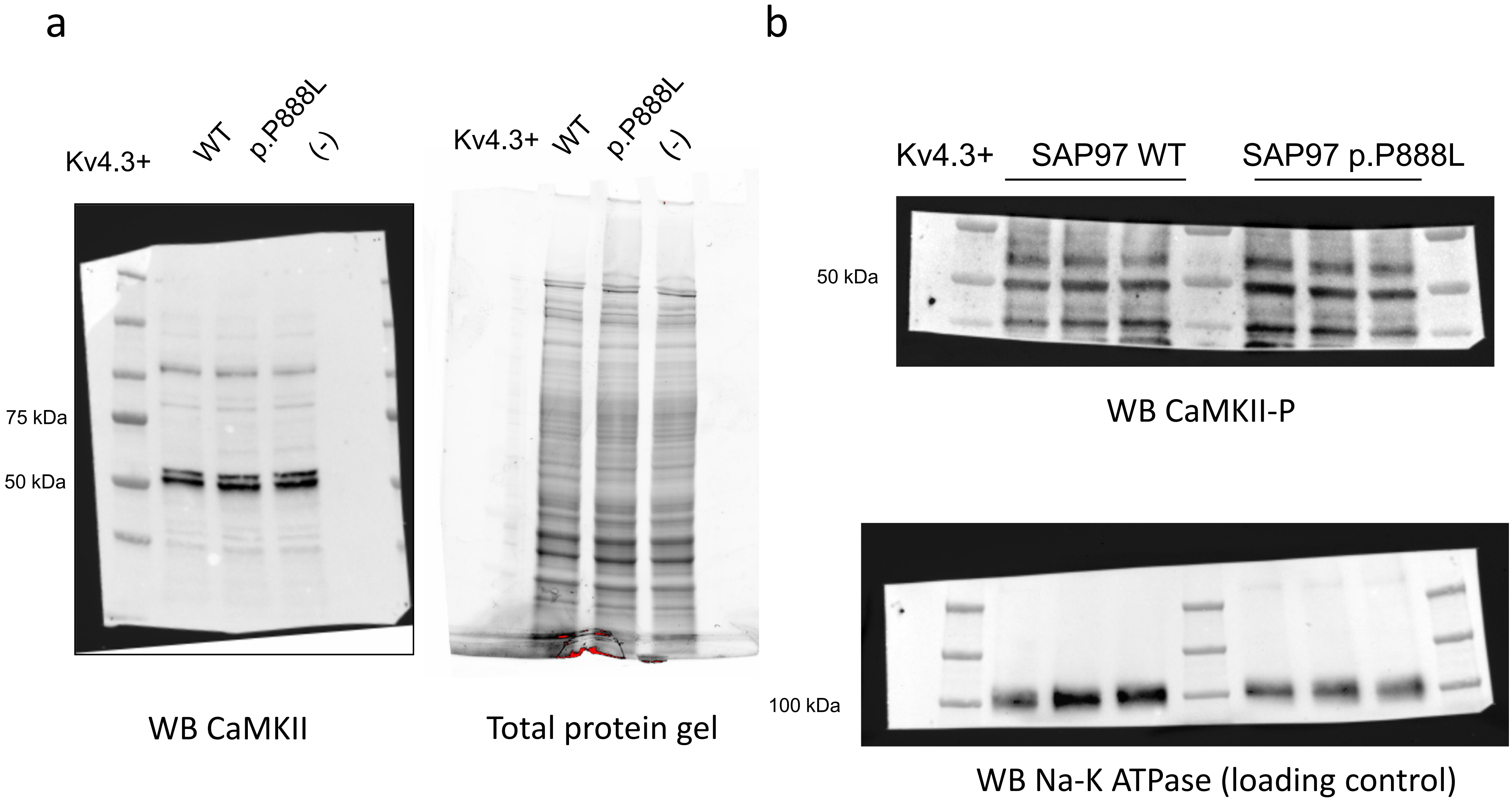

Supplementary Figure V

Supplementary Fig. VI. (a and b) Full length uncropped membranes and total protein gels of WB images shown in Fig. 7c (panel a) and 8c (panel b).

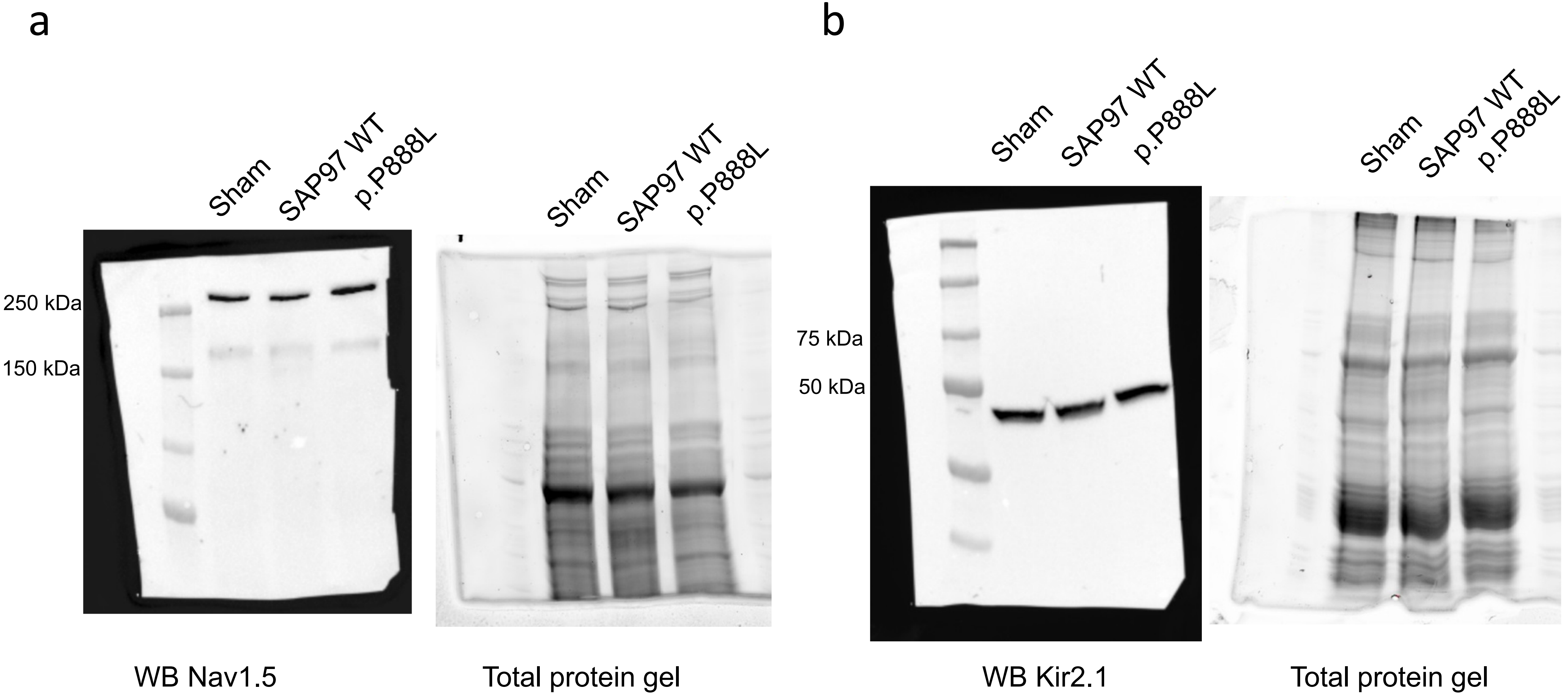

Supplementary Figure VI

Supplementary Fig. VII. Full length uncropped membrane and total protein gel of WB image shown in Supplementary Fig. III.

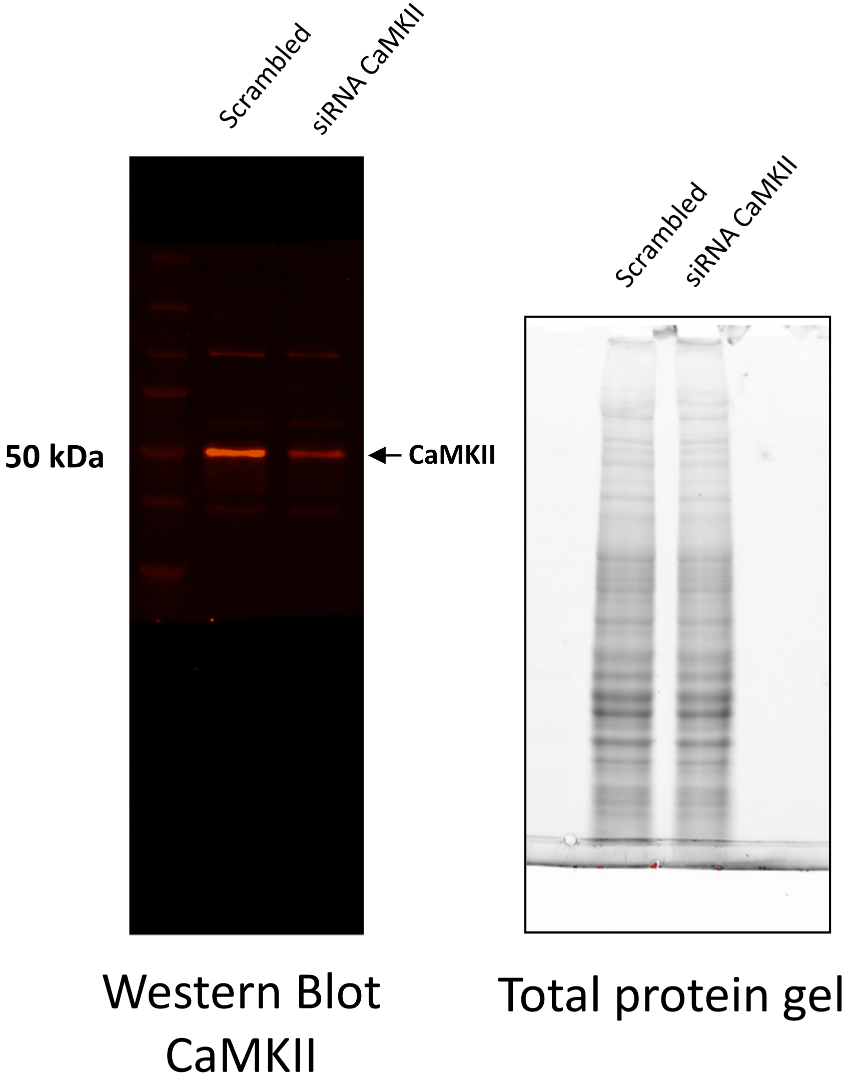

Supplementary Figure VII

Supplementary Table I. Prediction scores of the effect of the 5 most frequent missense *DLG1* variants on protein structure/function retrieved from SNP3d, Provean, and SIFT.

	dbSNP ID (ENST357674)	Total Allele Frequency (Percentage)	SNP3d			PROVEAN (-2.5)	SIFT (0.05)
			SVM profile	Entropy	PSSM score	Score	Score
p.R245Q	rs1134986	0.155 (15.5%)	1.65	1.89	2	-1.96	0.23
p.P888L	rs34492126	0.043 (4.3%)	-2.66	0.52	-5	-9.01	0.005
p.K140R	rs1802668	0.032 (3.2%)	0.93	1.62	1	-0.59	0.338
p.R786Q	rs78190191	0.017 (1.7%)	N/A	N/A	N/A	-2.98	0.045
p.D633E	rs35430440	0.007 (0.7%)	2.45	2.47	2	-1.06	0.175

dbSNP ID: reference number of each single nucleotide variant; N/A: not available; PROVEAN: Protein Variation Effect Analyzer; PSSM: Position Specific Scoring Matrix; SIFT: Sorting Intolerant From Tolerant; SVM: Support Vector Machine.
A negative SVM score indicates a deleterious substitution, considering that the larger the score, the more confident the classification. The higher the entropy score and PSSM scores are, the better tolerated is a mutation. PROVEAN scores classified the variants as deleterious if the cut-off value is ≤ -2.5 and as neutral if the cut-off value is ≥ -2.5. When the SIFT score is below the threshold (0.05) it is considered that the variant "affects protein function" and "tolerant" otherwise.

Up to now, 836 *DLG1* variants have been annotated in the Exome Aggregation Consortium database (http://exac.broadinstitute.org/). More than 50% of these variants are located in non-coding regions (intronic variants). Among the variants located in coding regions, up to 250 are non-synonymous or missense substitutions. We selected the 5 most frequent missense *DLG1* variants according to their total allele frequency value obtained from the Exome Aggregation Consortium database. We used 3 well recognized bioinformatics tools (SNP3d, PROVEAN, and SIFT) to predict the impact on the protein structure and function produced by these variants. The result of the analysis consistently shows that among the 5 most frequent missense *DLG1* variants, the p.P888L substitution is the most deleterious one i.e., the variant with the highest probability of changing structure, stability and function of the SAP97 protein.

Supplementary Table II. Time- and voltage-dependent properties of $I_{to,f}$, $I_{K,slow}$, $I_{Kv4.3}$, and $I_{Kv1.5}$.

			$I_{to,f}$ (Mouse cardiomyocytes)				
SAP97	V_{hact} (mV)	k_{act}	τ_f (ms)	τ (TEA) (ms)	V_{hinact} (mV)	k_{inact}	τ_{react} (ms)
Sham	-0.3±2.2	14.0±1.8	(-)	(-)	-45.8±2.2	10.4±0.8	28.4±5.7
WT	-1.4±1.6	17.0±1.1	49.0±4.3	40.2±3.9	-45.1±1.8	11.2±1.1	23.9±1.7
p.P888L	-6.2±2.2*#	17.6±1.3	68.2±4.1#	64.9±3.9#	-37.3±1.9*#	10.1±0.9	22.1±3.5<br>378±53
			$I_{Kv4.3}$ (CHO cells)				
SAP97	V_{hact} (mV)	k_{act}	τ_{finact} (ms)	τ_{sinact} (ms)	V_{hinact} (mV)	k_{inact}	τ_{react} (ms)
Sham	5.7±1.6	12.5±1.2	21.0±2.2	81.9±6.0	-38.9±2.6	5.9±0.2	159±20
WT	3.4±1.4	14.9±0.8	26.1±3.0*	128±15*	-42.0±1.9	5.3±0.5	194±14
p.P888L	-1.6±1.2*#	12.2±0.3	47.6±9.3*#	364±82*#	-30.4±2.7*#	6.1±0.2	419±68*
			$I_{Kv4.3}$ (CHO cells) + siRNA CaMKII				
SAP97	V_{hact} (mV)	k_{act}			V_{hinact} (mV)	k_{inact}	
WT	-2.5±2.9	14.9±1.1			-38.7±2.8	5.8±0.2	
p.P888L	-2.4±2.8	15.0±1.2			-39.2±3.1	5.7±0.3	
			$I_{Kv4.3}$ (CHO cells) + KN93				
SAP97	V_{hact} (mV)	k_{act}	τ_{finact} (ms)	τ_{sinact} (ms)	V_{hinact} (mV)	k_{inact}	
WT	-2.4±1.6	13.7±0.8	20.0±2.6#	87±8.2#	-35.7±2.0	5.4±0.3	
p.P888L	-2.1±1.6	14.9±0.6	27.3±3.1†	109±11†	-33.3±1.4	5.7±0.2	
		$I_{Kv1.5}$ (Ltk⁻ cells)				$I_{K,slow}$ (Mouse cardiomyocytes)	
SAP97	V_{hact} (mV)	k_{act}	τ_{inact} (ms)	τ_{deact} (ms)		τ_s (ms)	
Sham	-10.2±1.3	7.6±0.5	187±28	43.9±2.0		(-)	
WT	-11.9±1.8	6.2±0.6	226±40	51.9±5.8		1116±49	
p.P888L	-7.6±1.2	7.1±0.5	292±34	42.0±1.9		1251±44	

τ (TEA)= time constant of current inactivation yielded by the fit of a monoexponential function to the K^+ current traces recorded by applying 4-s pulses to +50 mV in the presence of 25 mM TEA. τ_{deact} = time constant of deactivation yielded by the fit of a monoexponential function to the tail $I_{Kv1.5}$ traces recorded at -40 mV after 500-ms pulses to +60 mV. τ_f and τ_s= fast ($I_{to,f}$) and slow ($I_{K,slow}$) time constants of current

inactivation yielded by the fit of a biexponential function to the K^+ current traces recorded by applying 4-s pulses to +50 mV. τ_{finact} and τ_{sinact}= fast and slow time constants of current inactivation yielded by the fit of a biexponential function to the $I_{Kv4.3}$ traces recorded by applying 500-ms pulses to +50 mV; τ_{inact} = time constant of inactivation yielded by the fit of a monoexponential function to the $I_{Kv1.5}$ traces recorded by applying 500-ms pulses to +60 mV. τ_{react} = time constant of recovery from inactivation for $I_{to,f}$ and $I_{Kv4.3}$. V_{hact} and k_{act}= midpoint and slope values of conductance-voltage ($I_{to,f}$, $I_{Kv4.3}$) or activation ($I_{Kv1.5}$) curves; V_{hinact} and k_{inact}= midpoint and slope values of the inactivation curves ($I_{to,f}$, $I_{Kv4.3}$). Each value represents mean±SEM of >6 experiments in each group. *P<0.05 vs Sham; #P<0.05 vs SAP97 WT; †P<0.05 vs p.P888L SAP97.

Supplementary Table III. Results of the blind-docking study of SAP97 and CaMKIIδ.

	Cluster rank	Number of conformations in the cluster	ΔG (kcal/mol) Center
SAP97 WT	0	50	-756.2
	1	46	-648.9
	2	41	-620.9
	3	38	-748.2
	4	37	-775.6
SAP97 p.P888L	0	55	-750.7
	1	47	-652.6
	2	43	-622.6
	3	38	-746.9
	4	37	-777.2

Supplementary Table IV. Time- and voltage-dependent properties of the I_{Na} recorded in mouse ventricular myocytes.

	I_{Na}							
SAP97	τ_{act} (ms)	V_{hact} (mV)	k_{act}	τ_{finact} (ms)	τ_{sinact} (ms)	V_{hinact} (mV)	k_{inact}	τ_{react} (ms)
Sham	0.5±0.07	-53.1±1.2	4.2±0.6	1.3±0.2	4.5±1.0	-87.6±5.3	4.9±0.5	8.2±1.7
WT	0.7±0.1	-53.6±0.8	4.0±0.4	1.3±0.1	6.4±1.7	-87.2±1.3	5.0±0.4	6.1±1.2
p.P888L	0.6±0.1	-53.0±1.2	4.4±0.5	1.8±0.2	6.2±1.3	-89.6±0.9	4.9±0.2	7.3±1.1

τ_{act}= time constant of activation measured at peak maximum current; τ_{finact} and τ_{sinact} = fast and slow time constants of current inactivation measured at peak maximum current; τ_{react} = time constant of recovery from inactivation; V_{hact} and k_{act}= midpoint and slope values of conductance-voltage curves; V_{hinact} and k_{inact}= midpoint and slope values of the inactivation curves. Each value represents mean±SEM of >6 experiments in each group.

REFERENCES

1. El-Haou, S. *et al.* Kv4 potassium channels form a tripartite complex with the anchoring protein SAP97 and CaMKII in cardiac myocytes. *Circ Res.* 104, 758-769, (2009).

2. Caballero, R. *et al.* Tbx20 controls the expression of the *KCNH2* gene and of hERG channels. *Proc Natl Acad Sci USA.* 114, E416-E425, (2007).

3. Matamoros, M. *et al*. Nav1.5 N-terminal domain binding to α1-syntrophin increases membrane density of human Kir2.1, Kir2.2 and Nav1.5 channels. *Cardiovasc Res.* 110, 279-290, (2016).

4. Sergeant, G. P. *et al*. Regulation of Kv4.3 currents by Ca^{2+}/calmodulin-dependent protein kinase II. *Am J Physiol Cell Physiol.* 288, C304-C313, (2005).

5. Cruz, F. M. *et al*. Exercise triggers ARVC phenotype in mice expressing a disease-causing mutated version of human plakophilin-2. *J Am Coll Cardiol.* 65, 1438-1450, (2015).

6. Gómez, R. *et al*. Nitric oxide inhibits Kv4.3 and human cardiac transient outward potassium current (I_{to1}). *Cardiovasc Res.* 80, 375-384, (2008).

7. Vaquero, M., Caballero, R., Gómez, R., Núñez, L., Tamargo, J. & Delpón, E. Effects of atorvastatin and simvastatin on atrial plateau currents. *J Mol Cell Cardiol.* 42, 931-945, (2007).

8. Pérez-Hernández, M. *et al*. Brugada syndrome trafficking-defective Nav1.5 channels can trap cardiac Kir2.1/2.2 channels. *JCI Insight.* 3, 96291, 10.1172/jci.insight.96291 (2018).

9. Utrilla, R. G. *et al*. Kir2.1-Nav1.5 Channel Complexes Are Differently Regulated than Kir2.1 and Nav1.5 Channels Alone. *Front Physiol.* 8, 903, 10.3389/fphys.2017.00903 (2017)

10. Barana, A. *et al*. Endocannabinoids and cannabinoid analogues block cardiac hKv1.5 channels in a cannabinoid receptor-independent manner. *Cardiovasc Res.* 85, 56-67, (2010).

11. Delpón, E. *et al*. Functional effects of KCNE3 mutation and its role in the development of Brugada syndrome. *Circ Arrhythm Electrophysiol.* 1, 209-218, (2008).

12. Dolz-Gaitón, P. *et al*. Functional characterization of a novel frameshift mutation in the C-terminus of the Nav1.5 channel underlying a Brugada syndrome with variable expression in a Spanish family. *PLoS One.* 8, e81493. 10.1371/journal.pone.0081493 (2013).

13. Caballero, R. *et al*. In humans, chronic atrial fibrillation decreases the transient outward current and ultrarapid component of the delayed rectifier current differentially on each atria and increases the slow component of the delayed rectifier current in both. *J Am Coll Cardiol.* 55, 2346-2354, (2010).

14. Moreno, I. *et al*. Effects of irbesartan on cloned potassium channels involved in human cardiac repolarization. *J Pharmacol Exp Ther.* 304, 862-873, (2003).

15. Núñez, L. *et al*. p.D1690N Nav1.5 rescues p.G1748D mutation gating defects in a compound heterozygous Brugada syndrome patient. *Heart Rhythm.* 10, 264-272, (2013)

16. Pérez-Hernández, M. *et al*. Pitx2c increases in atrial myocytes from chronic atrial fibrillation patients enhancing I_{Ks} and decreasing $I_{Ca,L}$. *Cardiovasc Res.* 109, 431-441, (2016).

17. Xu, H., Guo, W. & Nerbonne, J.M. Four kinetically distinct depolarization-activated K^+ currents in adult mouse ventricular myocytes. *J Gen Physiol.* 113, 661-678, (1999).

18. Kozakov, D. *et al*. The ClusPro web server for protein-protein docking. *Nat Protoc.* 12, 255-278, (2017).

19. Zhu, J., Shang, Y., Xia, C., Wang, W., Wen, W. & Zhang, M. Guanylate kinase domains of the MAGUK family scaffold proteins as specific phospho-protein-binding modules. *EMBO J.* 30, 4986-4997, (2011).
